# Disrupting PDGFRA-driven immune evasion in glioma: vaccine-based strategies on the horizon

**DOI:** 10.3389/fonc.2026.1666612

**Published:** 2026-03-02

**Authors:** Xialin Zhang, Xinwei Li, Ran Cui, Xinlin Yu, Zihan Zhang, Zhongxiang Luo, Gang Chen, Sheng Lin

**Affiliations:** 1 Department of Oncology, Affiliated Hospital of Southwest Medical University, Luzhou, Sichuan, China; 2 Department of Respiratory and Critical Care Medicine, First People’s Hospital of Neijiang, Neijiang, Sichuan, China; 3 Department of Oncology, Affiliated Hospital of Chengdu University, Chengdu, Sichuan, China; 4 Department of General Surgery (Hepatopancreatobiliary Surgery), Affiliated Hospital of Southwest Medical University, Luzhou, Sichuan, China

**Keywords:** glioma, immune escape, immunotherapy, microenvironment, PDGFRA, personalized medicine, vaccine therapy

## Abstract

Gliomas, the most aggressive primary brain tumors, present a formidable challenge in neuro-oncology, characterized by infiltrative growth, high recurrence rates, and a profoundly immunosuppressive microenvironment that severely limits the efficacy of current treatments. Platelet-derived growth factor receptor alpha (PDGFRA) has emerged as a pivotal oncogenic driver in gliomas, not only promoting cellular proliferation and angiogenesis but critically orchestrating complex immune evasion mechanisms. Understanding how PDGFRA shapes this immunosuppressive landscape is paramount for developing effective immunotherapies, especially given the minimal response rates of gliomas to conventional checkpoint inhibitors. PDGFRA signaling actively remodels the glioma microenvironment, contributing to vascular abnormalities (e.g., via the PDGFRA-Endocan-MYC axis), metabolic reprogramming that impairs T cell function, and immune cell polarization, all of which restrict anti-tumor immunity. Crucially, vaccine-based therapeutic modalities targeting PDGFRA offer a compelling dual strategy: they hold the potential to suppress tumor proliferation while simultaneously reversing immune evasion mechanisms. This positions PDGFRA-targeted vaccines as a significant innovation on the horizon for glioma immunotherapy. Addressing substantial translational hurdles, including blood-brain barrier impermeability, inherent tumor heterogeneity, and the pervasive immunosuppressive milieu, is essential for clinical success. Future clinical translation will require the integration of multi-omics to identify immunogenic neoantigens, the implementation of advanced nanodelivery systems for optimized vaccine distribution and efficacy, and synergistic combinations with immune checkpoint inhibitors to overcome resistance. By dissecting the intricate PDGFRA-mediated signaling network, we highlight critical targets and outline strategies for developing precision-oriented, individualized immunotherapeutic interventions, aiming to significantly improve outcomes for patients with gliomas.

## Introduction

Gliomas represent a formidable challenge in neuro-oncology, with poor survival outcomes despite multimodal treatment approaches (1). Their therapeutic resistance stems from infiltrative growth patterns and sophisticated immune evasion mechanisms that create a profoundly “cold” tumor microenvironment (2, 3). Platelet-derived growth factor receptor alpha (PDGFRA) has emerged as a critical oncogenic driver in gliomas (4). Beyond promoting cellular proliferation and angiogenesis, PDGFRA orchestrates complex immune evasion mechanisms, positioning it at the intersection of oncogenic signaling and immunosuppression (5). Consequently, understanding and disrupting PDGFRA-mediated immune evasion holds significant promise for the development of novel, translational immunotherapeutic interventions for this challenging disease.

Immunotherapy has transformed cancer treatment paradigms, yet its efficacy in gliomas has been markedly limited, with checkpoint inhibitor response rates generally remaining below 10% (6). This resistance stems from a complex interplay of factors, including the intrinsic impermeability of the blood-brain barrier (BBB), a generally low tumor mutational burden, and a profoundly immunosuppressive tumor microenvironment. This microenvironment is characterized by the abundance of immunosuppressive cells, such as regulatory T-cells and myeloid-derived suppressor cells, alongside M2-polarized tumor-associated macrophages (7). Crucially, PDGFRA activation plays a pivotal role in shaping this adverse immune landscape. Through intricate mechanisms, PDGFRA signaling contributes to the modulation of cytokine networks, orchestrates metabolic reprogramming that impairs T-cell effector functions, and promotes vascular remodeling that restricts essential immune cell infiltration into the tumor core (8, 9). Consequently, targeting PDGFRA presents a compelling therapeutic opportunity, as it may simultaneously inhibit oncogenic signaling driving tumor progression while actively reversing the tumor-mediated immunosuppression that thwarts effective anti-tumor immunity (10, 11).

This review examines PDGFRA’s multifaceted role in glioma pathophysiology, emphasizing its immunomodulatory functions. We delineate PDGFRA signaling networks and explore how it orchestrates immune evasion through three critical mechanisms: vascular remodeling, immune cell polarization, and metabolic reprogramming. We assess current PDGFRA-targeted interventions, including small molecule inhibitors and emerging vaccine-based approaches, critically evaluate translational challenges, and outline promising directions in personalized immunotherapy. Through this analysis, we provide a framework for developing PDGFRA-targeted interventions that may overcome the formidable challenge of immune evasion in gliomas.

## Role of PDGFRA signaling pathway in gliomas

### Structure, function, and dysregulation of PDGFRA in gliomas

PDGFRA is a transmembrane receptor tyrosine kinase (RTK) and a member of the type III RTK family, playing a critical role in biological processes, particularly during development. Physiologically, PDGFRA is crucial for neural development, governing the proliferation and differentiation of neural progenitor cells, with a notable essential role in the development of oligodendrocyte progenitor cells (OPCs) (12). In the adult brain, PDGFRA expression significantly diminishes, being primarily confined to a subset of progenitor cells and perivascular cells where it contributes to neuroglia homeostasis and the maintenance of BBB integrity (13). However, during pathological conditions, including neuronal injury and glioma, PDGFRA expression is substantially upregulated, initiating downstream signaling cascades. This upregulation exhibits pronounced spatiotemporal specificity, with a particular prevalence observed in neuroglial progenitor cells and astrocytes (14). Aberrant activation of PDGFRA is a hallmark of many gliomas, driven by mechanisms such as gene amplification or specific mutations (e.g., D842V, V561D) that lead to conformational changes in the kinase domain, resulting in ligand-independent, persistent activation (5).

PDGFRA signaling activation primarily occurs through ligand-dependent and ligand-independent pathways. In ligand-dependent activation, PDGF ligand binding induces receptor dimerization, precipitating autophosphorylation of the intracellular kinase domain. The phosphorylated receptor subsequently recruits diverse downstream signaling molecules (5, 15). For instance, within hypoxic microenvironments, PDGF ligand expression and secretion are regulated by the transcription factor HIF-1α; concurrently, the downstream signaling molecule PTEN negatively regulates the PI3K/AKT pathway (5). Ligand-independent activation mechanisms have also been identified in gliomas. These include epigenetic modifications such as altered histone modifications and DNA methylation influencing PDGFRA gene expression (16). Furthermore, diminished miR-34a expression leads to enhanced PDGFRA mRNA stability (17) and cytokine signaling, such as IL-6 secreted by tumor-associated macrophages (TAMs), can augment PDGFRA expression via STAT3-mediated positive feedback loops (18). The dysregulation of these regulatory mechanisms in gliomas results in the aberrant activation of the PDGFRA signaling pathway. It is important to note that co-activation of PDGFRA and EGFR can trigger compensatory resistance in signaling pathways, potentially limiting the efficacy of therapies targeting only a single pathway (19).

Following activation, PDGFRA principally transduces signals through three critical signaling cascades: initially, the PI3K/AKT/mTOR pathway drives cellular survival and metabolic reprogramming; subsequently, the RAS/RAF/MEK/ERK signaling cascade propels cellular proliferation and differentiation processes; finally, the JAK/STAT pathway mediates the regulation of gene transcription and inflammatory responses (20–22). Additionally, recent discoveries indicate that PDGFRA can participate in tumor immune evasion mechanisms via non-canonical pathways, such as direct interaction with PD-L1 (23). The regulation of these signaling pathways encompasses multi-tiered mechanisms, including dephosphorylation mediated by protein tyrosine phosphatases (PTPs), receptor internalization and degradation, miRNA-regulated expression, and crosstalk with other RTKs (e.g., EGFR) (24).

**Figure 1:**
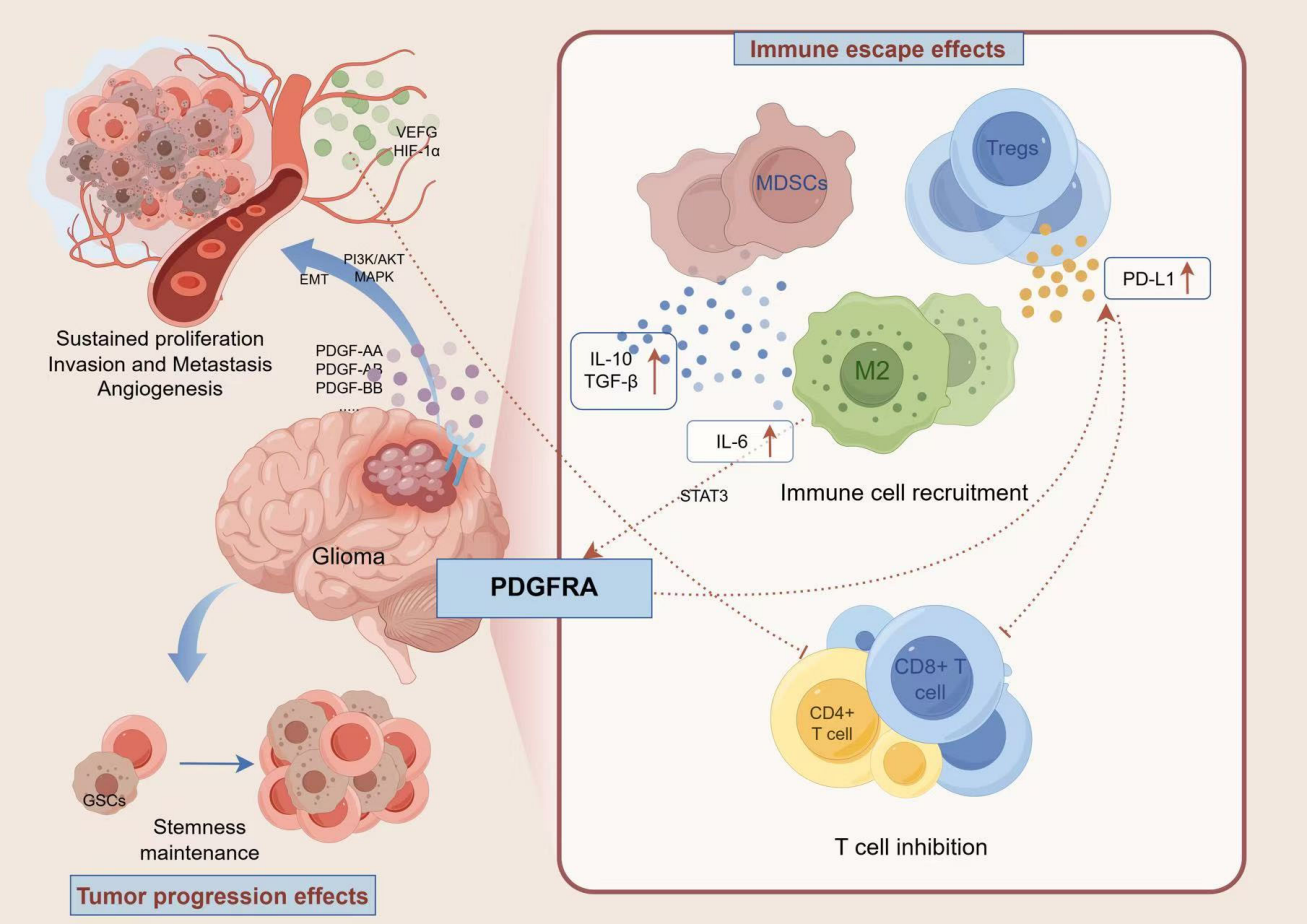
PDGFRA signaling: A dual orchestrator of glioma progression and immune evasion. PDGFRA is aberrantly activated in gliomas due to gene amplification, mutations (such as D842V), or extracellular domain deletion mutations. It drives cell proliferation, metabolic reprogramming, and EMT-like invasion by activating downstream signaling pathways including PI3K/AKT and RAS/RAF/MEK. Meanwhile, PDGFRA upregulates PD-L1, recruits immunosuppressive cells (e.g., regulatory T cells), and downregulates MHC-I expression to establish an immunosuppressive microenvironment. It also mediates immune evasion through direct interaction with PD-L1. Genetic variations of PDGFRA (amplification, activating mutations, fusions) are of critical significance in molecular subtyping of gliomas, particularly enriched in highly malignant subtypes.

PDGFRA genetic alterations manifest in diverse forms, predominantly comprising gene amplification (approximately 15%), activating mutations (approximately 5-7%), and rare KIT-PDGFRA fusions (25). The fifth edition of the WHO Classification of Central Nervous System tumors, published in 2021, incorporates molecular characteristics as essential elements in glioma classification, with PDGFRA genetic alterations constituting a pivotal feature in molecular subtyping. PDGFRA alterations exhibit heterogeneous distribution across glioma subtypes. Within glioblastomas, approximately 26% of IDH-wildtype cases and 11% of IDH-mutant cases demonstrate PDGFRA overexpression or genetic alterations (26). Single-cell sequencing and spatial transcriptomic analyses have revealed PDGFRA alterations predominantly enriched in “proneural” and “mesenchymal” molecular subtypes, which typically manifest heightened malignancy and inferior prognostic outcomes (27). PDGFRA exhibits complex interactions with classical glioma genetic markers, displaying significant co-occurrence or mutual exclusivity relationships with IDH1/2 mutations, 1p/19q codeletion, and MGMT promoter methylation. Notably, PDGFRA amplification occurs more frequently in IDH-wildtype glioblastomas and typically demonstrates mutual exclusivity with EGFR amplification (28). PDGFRA mutations (e.g., D842V, R841K) are detected in approximately 5-7% of gliomas, predominantly localized to the intracellular kinase domain, resulting in constitutive receptor activation (29). Additionally, 4q12 amplifications involving PDGFRA, KIT, and KDR genes have been identified in a subset of gliomas. Significantly, a distinct subgroup of pediatric high-grade gliomas—diffuse intrinsic pontine glioma (DIPG)—frequently harbors PDGFRA mutations or fusions, co-occurring with epigenetic abnormalities such as H3K27M, indicating complex molecular pathogenetic mechanisms (30). Multi-omic analyses based on the Gene Expression Omnibus (GEO) and The Cancer Genome Atlas (TCGA) databases demonstrate that PDGFRA-altered gliomas exhibit distinctive metabolic reprogramming characteristics (enhanced glutamine dependency), potentially serving as actionable therapeutic targets and providing rationale for personalized treatment approaches (31, 32) (**Figure 1**).

### PDGFRA-mediated immune evasion

The PDGFRA signaling pathway is a critical regulator in glioma pathogenesis, implicated in tumor initiation, progression, and importantly, in orchestrating immune evasion. Upregulation of PDGFRA is observed across various aggressive glioma subtypes, including diffuse midline gliomas and pediatric high-grade gliomas, acting as a key driver in the transition from low-grade to high-grade disease (33). Notably, extracellular domain deletion mutations in PDGFRA enhance its synergistic interaction with immunosuppressive cytokines, such as TGF-β,within the tumor microenvironment, thereby facilitating immune evasion (30). During tumor initiation, PDGFRA promotes cellular proliferation and survival, partly by activating the PI3K/AKT and MAPK pathways, which upregulate anti-apoptotic proteins (e.g., Bcl-2, Bcl-xL) and enhance cell cycle progression (34). Single-cell sequencing data reveal a strong correlation between high PDGFRA expression in glioma cells and T cell exhaustion phenotypes (35).

PDGFRA signaling recruits immunosuppressive cells, including regulatory T cells (Tregs), TAMs, and myeloid-derived suppressor cells (MDSCs). These recruited cells, in turn, secrete immunosuppressive cytokines such as IL-10 and TGF-β. PDGFRA can also downregulate MHC-I expression on tumor cells, collectively creating an immunosuppressive milieu that blunts anti-tumor immune responses (36). This profound impact on the immune landscape provides a strong rationale for targeting PDGFRA in the development of effective glioma vaccine strategies.

## PDGFRA-mediated tumor immune microenvironment remodeling

### PDGFRA’s core role in regulating the immune microenvironment

PDGFRA critically orchestrates tumor-immune cell interactions through multiple signaling pathways, significantly shaping the tumor immune microenvironment (TME). Within neoplastic cells, PDGFRA signaling upregulates chemokines like CCL2 and CCL5, which promote monocyte migration and M2-like polarization (37). Transcriptomic analysis of glioblastoma (GBM) with PDGFRA alterations reveals enrichment in immune evasion pathways, including the downregulation of Major Histocompatibility Complex (MHC) class I molecules and the upregulation of immune checkpoint ligands such as PD-L1 (38). Crucially, PDGFRA plays a multifaceted role in fostering an immunosuppressive tumor microenvironment (TME) that hinders therapeutic efficacy, including immune-based interventions. PDGFRA overexpression has been linked to increased PD-L1 expression on glioma cells, leading to suppressed CD8+ T cell activity. It also enhances the self-renewal and differentiation capacity of glioma stem cells (GSCs), contributing to chemo- and radioresistance (39, 40).

Furthermore, in patient-derived GBM models, the PDGFA-PDGFRA signaling axis elevates expression of the tryptophan-degrading enzyme indoleamine 2,3-dioxygenase 1 (IDO1). This enzymatic activity depletes tryptophan, a crucial nutrient for T cell function, thereby suppressing T cell activity (41). Concurrently, PDGFRA-mediated angiogenesis contributes to dysregulated vascular architecture, impeding immune cell infiltration. For instance, in models where Endocan, a proteoglycan secreted by endothelial cells, activates PDGFRA, it enhances vascular permeability and fosters hypoxic microenvironments (42). These hypoxic regions further drive HIF-1α-dependent secretion of VEGF and TGF-β, thereby intensifying immunosuppression (43).

Clinical data from TCGA demonstrates that GBM exhibiting high PDGFRA expression manifest reduced CD8+ T cell infiltration, elevated expression of immunosuppressive cytokines, and correlate with inferior survival outcomes (44). Notably, the co-expression of PDGFRA with the receptor tyrosine kinase EphA2 exacerbates this phenotype by promoting epithelial-mesenchymal transition (EMT) and further attenuating T cell chemotaxis. Collectively, these observations highlight PDGFRA’s central role as a regulator of tumor microenvironment architecture, integrating intrinsic tumor cell signaling with systemic immune dysregulation.

### PDGFRA-driven vascular remodeling and the dual roles of blood and lymphatic vessels

PDGFRA-driven vascular remodeling in gliomas creates barriers to immune infiltration. Endocan (Esm1) from endothelial cells activates tumor PDGFRA, triggering MYC-dependent vascular sprouting, basement membrane changes, and leaky vessels with poor pericyte coverage, impairing perfusion and immune extravasation (42, 45). Esm1 knockout reduces permeability and boosts T cell infiltration (46). Genomic analysis reveals that PDGFRA is frequently co-amplified with the vascular regulator KDR (VEGFR2) and KIT at the 4q12 locus in high-grade gliomas, creating a dependency on this oncogenic axis. Targeting this niche with avapritinib, a highly CNS-penetrant PDGFRA/KIT inhibitor, demonstrated significant efficacy in a clinical cohort of pediatric patients (n=8) harboring PDGFRA amplifications or exon 18 mutations, achieving objective radiographic responses in 42% of evaluable cases (45). Mechanistically, PDGFRA inhibition can indirectly reduce VEGF secretion and stabilize vascular structure by inhibiting the PDGFRA signaling axis.

PDGFRA-mediated angiogenesis has bidirectional effects. Pathological angiogenesis forms immunosuppressive barriers, hinders drug delivery, and aids tumor progression/metastasis; vascular normalization improves tumor perfusion, immune cell infiltration, and therapeutic synergy (47, 48). Angiogenesis and lymphangiogenesis collectively exert dual roles. Normalized blood vessels enhance drug delivery efficiency and therapeutic efficacy, while aberrant angiogenesis drives tumor growth and drug resistance. The crosstalk between blood vessels and lymphatics modulates this process, either clearing tumor-derived factors or facilitating tumor metastasis through the PDGFRA/VHL/HIF-VEGF signaling pathway (49, 50). Monotherapy prompts bypass (VEGFR2/FGFR1 upregulation), lymphangiogenic compensation (VEGF-C/PDGF-BB), and immune escape. Vaccines or combination therapies should co-target pathway redundancies or lymphangiogenesis to prevent drug resistance.

Unlike anti-VEGF therapies that exacerbate tumor hypoxia while suppressing angiogenesis, PDGFRA inhibition induces vascular normalization by disrupting MYC signaling, thereby improving tumor perfusion and anti-tumor immunity. PDGFRA vaccines outperform EGFRvIII vaccines due to their broad expression profile, dual effects of oncogenic pathway disruption and immune modulation, and lower escape rate; they show promise despite tumor heterogeneity, though further clinical validation is needed.

### PDGFRA signaling: sculpting immune cell polarization and function in the tumor microenvironment

PDGFRA signaling pathways critically influence the differentiation and functional polarization of immune cells within the tumor microenvironment. In myeloid lineage cells, PDGFRA activation promotes differentiation into MDSCs. These MDSCs suppress T cell responses by producing nitric oxide and arginase (51). Notably, in a PDGFRA-driven glioblastoma murine model, eliminating PDGFRA-expressing myeloid cells led to enhanced T cell effector functions and extended survival (52).

Within the tumor microenvironment, high PDGFRA expression in tumors correlates with a prevalent M2-like phenotype in TAMs, characterized by increased CD206 expression and reduced inflammatory markers. This M2 polarization is promoted by PDGFRA-driven secretion of M-CSF and IL-10 from tumor cells. Co-culture experiments have demonstrated that inhibiting PDGFRA can reverse these M2 phenotypic markers (53).

**Figure 2:**
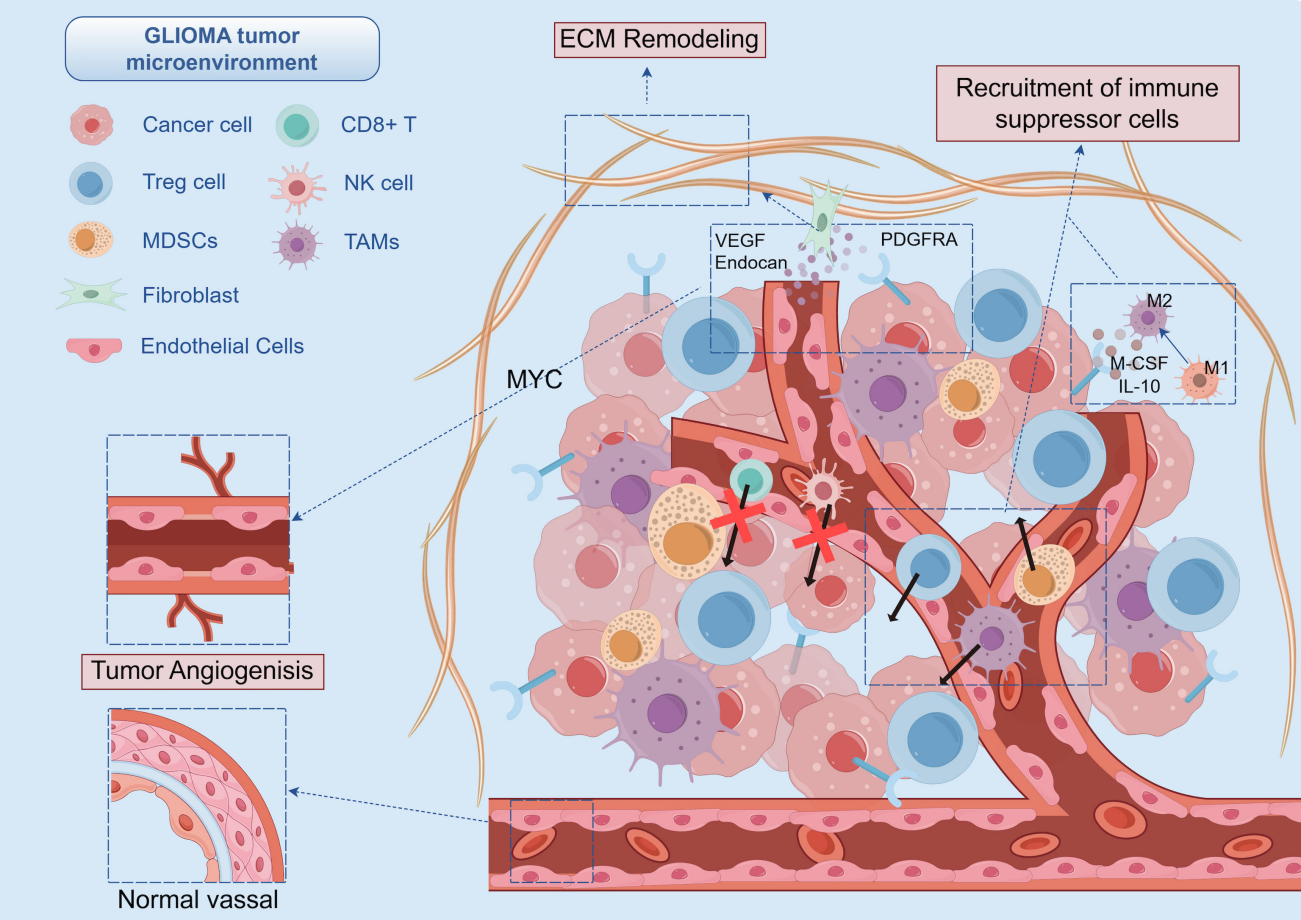
PDGFRA-mediated remodeling of glioma microenvironment: Vascular abnormalities and immune cell exclusion. PDGFRA drives angiogenesis sprouting and basement membrane remodeling in gliomas via the Endocan-PDGFRA-MYC axis, forming immature vascular networks. This not only hinders immune cell extravasation but also reduces vascular stability by decreasing pericyte coverage. Meanwhile, PDGFRA activation in pericytes promotes their differentiation into myofibroblasts, which secrete extracellular matrix to form a physical barrier. Additionally, PDGFRA signaling directly promotes myeloid cell differentiation into MDSCs and polarizes TAMs toward an M2-like phenotype. It also impairs T cell effector functions and induces exhaustion through binding to PDGFA ligands.

For T cells, the binding of soluble ligands like PDGFA to PDGFRA impairs effector differentiation and drives T cell exhaustion. PDGFA-exposed CD8^+^T cells exhibit reduced IFN-γ production and increased PD-1 expression, a phenotype that can be restored by PDGFRA blockade (54). This impairment of T cell function is particularly severe in tumors with PDGFRA/EPHA2 co-amplification, where combined receptor signaling further exacerbates T cell dysfunction (55) (**Figure 2**).

## Regulatory interactions between PDGFRA signaling and the metabolism-immune axis

### PDGFRA signaling integrates metabolic reprogramming and immunosuppression

PDGFRA functions as a key receptor tyrosine kinase, integrating cellular metabolism and immune responses. In glioblastoma specimens with high PDGFRA expression, genes associated with glycolysis (e.g., HK2, LDHA) are coordinately upregulated with immune checkpoint molecules (e.g., PD-L1, CTLA-4) (42). Mechanistically, PDGFRA phosphorylation activates AKT, which subsequently phosphorylates mTORC1. This cascade promotes the translation of glucose transporter GLUT1 via S6K1, while simultaneously inhibiting mitochondrial oxidative phosphorylation. This metabolic shift not only enhances tumor cell proliferation but also suppresses T cell function by creating a locally glucose-depleted and lactate-rich environment (56, 57).

PDGFRA signaling additionally modulates tumor microenvironment remodeling via microglia-mediated immune interactions. In IDH-mutant gliomas, the aberrantly activated PDGFRA-SHP2 axis synergizes with RAS-MAPK signaling to drive PD-L1 overexpression. This activation reprograms immune cell phenotypes and dampens therapeutic efficacy, establishing a “signaling-immunosuppression” feedback loop targetable by bionic nanoplatforms (45, 58–60). Studies using patient-derived IDH-mutant glioma stem cells (GSCs) demonstrate that the SHP2 inhibitor SHP099 can reverse PDGFRA-mediated MYC phosphorylation. This intervention restores oxidative phosphorylation, reduces IL-10 secretion, and reactivates cytotoxic function in CD8^+^ T cells (58). Recent studies reveals that PDGFRA^+^ tumor cell subpopulations exhibit co-expression networks between glycolysis gene modules and M2 macrophage polarization markers (such as CD163 and MRC1), suggesting metabolic reprogramming directly orchestrates myeloid cell phenotypes (55).

### Regulatory role of the PDGFRA-endocan-MYC axis in the vascular-metabolic microenvironment

Endocan, secreted by tumor vascular endothelial cells, regulates tumor vascularity and spatial phenotype via direct binding and activation of PDGFRA on glioblastoma cells (46). Endocan-PDGFRA signaling activates downstream PI3K and ERK pathways and enhances chromatin accessibility at the Myc promoter, resulting in stable upregulation of MYC expression (61). This signaling axis is essential for establishing the hypervascular phenotype characteristic of GBM and confers radioprotection, consistent with the role of the perivascular niche in therapeutic resistance (62, 63). Distinct from mechanisms promoting immune exclusion (no significant differences in CD8+ T cell infiltration were observed between Esm1 wild-type and knockout tumors), the Endocan-PDGFRA-MYC axis primarily functions to maintain GBM cells in a proliferative, proneural state at the perivascular niche, thereby preventing the stress-induced mesenchymal differentiation typically observed in the tumor core (42, 64, 65).

PDGFRA-mediated tumor-vascular crosstalk represents a critical determinant of glioblastoma progression and therapeutic resistance (66), primarily manifested through its regulation of intratumoral heterogeneity and plasticity rather than direct immune suppression. Investigations demonstrate that the Endocan-PDGFRA axis supports a reciprocal feedback loop where endothelial-secreted factors drive GBM proliferation and promote further neovascularization. Consequently, inhibition of PDGFRA signaling (e.g., with ponatinib) effectively disrupts this vascular-tumor support system, downregulates MYC, and reduces tumor growth specifically in Endocan-rich microenvironments, highlighting the Endocan-PDGFRA axis as a potential therapeutic target to subdue GBM recurrence.

### Metabolic-immune reprogramming by the PDGFRA-SHP2 axis in IDH-mutant gliomas

Isocitrate dehydrogenase (IDH) mutations activate PDGFRA via epigenetic mechanisms, driving distinct metabolic-immune interaction patterns (67). IDH mutations result in accumulation of the oncometabolite D-2-hydroxyglutarate (D-2-HG), which competitively inhibits α-ketoglutarate (α-KG)-dependent dioxygenases, thereby disrupting epigenetic regulatory mechanisms and consequent gene expression profiles. These metabolic alterations not only promote tumorigenesis and progression but also enhance immunosuppressive states through modulation of the immune microenvironment.

The PDGFRA-SHP2 axis plays a pivotal role in this process (68). PDGFRA activation, via SHP2, further influences downstream signaling pathways, including ERK and PI3K/AKT/mTOR cascades, which are critical for neoplastic cell proliferation, survival, and metabolic reprogramming. Additionally, the PDGFRA-SHP2 axis promotes the establishment of an immunosuppressive microenvironment through upregulation of immune checkpoint molecules and suppression of genes associated with anti-tumor immune responses (including IFNA/B gene clusters) (58).

**Figure 3:**
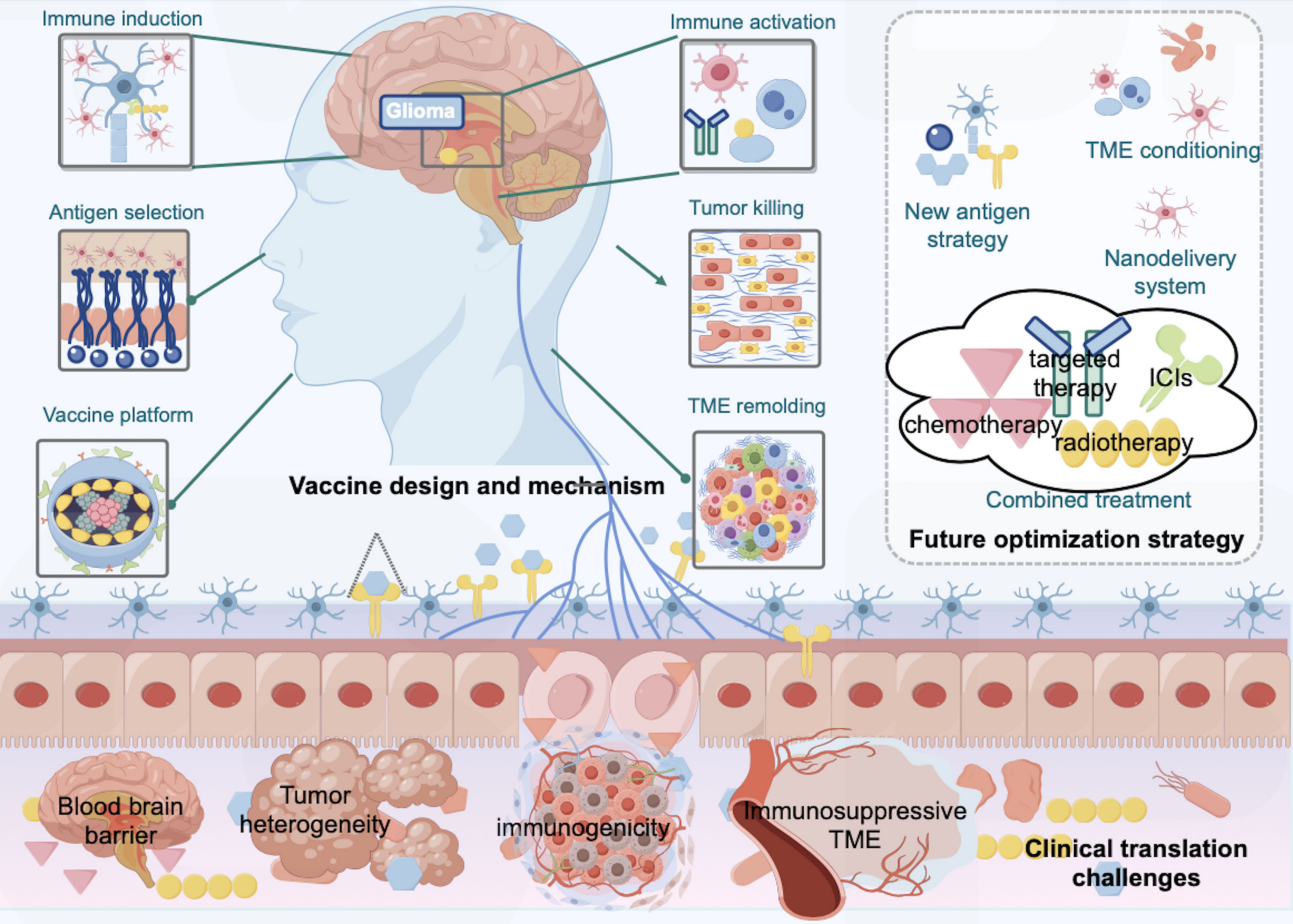
Crosstalk of PDGFRA signaling with metabolism-immunity axis: Unveiling Endocan-MYC and SHP2 pathways. In gliomas, PDGFRA drives the formation of an immunosuppressive metabolic microenvironment through dual signaling axes: In the PDGFRA-Endocan-MYC axis (left panel), PDGFRA activation promotes Endocan secretion from endothelial/tumor cells, triggering the integrin-MYC signaling cascade that enhances glycolysis (lactate accumulation) and glutaminolysis (kynurenine production), directly suppressing T cell activity and inducing Treg differentiation. In the PDGFRA-SHP2 axis (right panel, IDH-mutant context), PDGFRA activates SHP2 phosphatase, which synergizes with the oncometabolite 2-HG to disrupt mitochondrial metabolism (e.g., TCA cycle dysfunction), leading to CD8+ T cell exhaustion and PD-1 overexpression. These dual axes cooperatively induce metabolic competition imbalance, immune checkpoint activation, and ultimately the collapse of antitumor immunity, shaping an immune-privileged tumor microenvironment.

Metabolically, the PDGFRA-SHP2 axis enhances lactate dehydrogenase A (LDHA) expression, augmenting lactate production and consequent acidification of the tumor microenvironment (69). This acidic milieu not only suppresses T cell functionality but also promotes accumulation of immunosuppressive cells, notably MDSCs. Concurrently, the PDGFRA-SHP2 axis modulates expression of angiogenic factors, including VEGF-C and Angpt2, facilitating aberrant angiogenesis and further restricting immune cell infiltration (70) (**Figure 3**).

## PDGFRA-related vaccine development: foundations and prospects

### Challenges in glioma immunotherapy

The cardinal challenges in glioma immunotherapy comprise tumor heterogeneity-induced antigen escape and the profoundly immunosuppressive tumor microenvironment (71–73). Despite breakthrough advancements with immune checkpoint inhibitors (ICIs) and chimeric antigen receptor T cell (CAR-T) therapies across diverse solid malignancies, their efficacy remains substantially limited in glioma treatment (74). This therapeutic limitation is primarily attributable to several factors: pronounced molecular and cellular heterogeneity in gliomas, resulting in differential antigen expression profiles that complicate specific tumor antigen identification (75). Elevated expression of immunosuppressive molecules and enrichment of suppressive cellular populations within the glioma microenvironment, impeding effector T cell functionality (76, 77). And tumor cell immune evasion strategies, including downregulation of major histocompatibility complex (MHC) molecules and inhibition of antigen presentation mechanisms (78, 79).

The blood-brain barrier (BBB) further exacerbates the challenges in glioma immunotherapy. The BBB functions as a natural barrier for the central nervous system, stringently restricting the penetration of exogenous substances and immune cells into the brain parenchyma (80). Conventional immunotherapeutic agents encounter significant difficulty effectively traversing the BBB to reach tumor sites and exert their therapeutic effects (81). While disruption of BBB integrity may partially enhance drug penetration, intratumoral hypertension and cerebral oedema further constrain drug permeation into the tumor (82).

The high diversity and redundancy of tumor driver genes and signaling pathways constitute additional obstacles to glioma immunotherapy. Beyond PDGFRA, multiple critical genes—including EGFR, IDH1/2, TERT, PTEN, and TP53—play significant roles in glioma development and progression (44). These genetic mutations or copy number variations frequently co-exist and demonstrate complex regulatory interrelationships (75). PDGFRA functions not merely as an independent oncogenic driver but occupies a pivotal position within the regulatory network of multiple genetic abnormalities. Targeted intervention against PDGFRA may potentially disrupt tumor progression mechanisms driven by cooperative multi-gene mutations, representing a significant breakthrough opportunity for precision therapy in glioma.

Glioma stem cells (GSCs) present another significant challenge. GSCs play crucial roles in glioma initiation, progression, recurrence, and therapeutic resistance (83). These cells maintain their stemness through diverse mechanisms, including activation of signaling pathways such as PDGFRA, Notch, and Wnt, while establishing specialized metabolic and epigenetic regulatory environments (30, 84). Consequently, effective elimination of GSCs or induction of their differentiation represents a key determinant for enhancing immunotherapeutic efficacy.

Addressing these challenges, PDGFRA signaling pathway-related vaccine development represents a significant research direction for confronting the therapeutic dilemmas in glioma treatment. On one hand, the high-frequency mutations and specific expression of PDGFRA provide ideal targets for vaccine design (17, 33). Vaccines targeting PDGFRA mutation epitopes must effectively induce specific T cell responses while simultaneously activating both CD4^+^ and CD8^+^ T cells, potentially overcoming barriers imposed by tumor heterogeneity (45, 85). On the other hand, the close regulatory relationship between PDGFRA and PD-1/PD-L1 pathways provides a rational theoretical foundation for combination interventions with immune checkpoint inhibitors (60). Furthermore, PDGFRA’s association with tumor metabolism, angiogenesis, and GSC stemness maintenance offers additional possibilities for multi-target combination vaccine designs (86). Consequently, the development of optimal PDGFRA vaccines holds promise for surmounting multiple barriers in glioma immunotherapy.

### Basic principles of tumor vaccine design

Vaccine therapy represents a transformative breakthrough in glioma treatment, necessitating consideration of multiple factors including target antigen determination, vaccine platform selection, delivery systems, and adjuvant utilization. Diverse vaccine modalities present distinct advantages and limitations, encompassing peptide vaccines, dendritic cell (DC) vaccines, and DNA/RNA vaccines, among others.

Ideal tumor vaccines require highly immunogenic antigens that are exclusively expressed in tumor cells and essential for their survival (87). PDGFRA vaccine design focuses on recognizing and targeting tumor-specific antigens (TSAs) and tumor-associated antigens (TAAs) in glioma cells to stimulate immune responses directed against neoplastic cells (88–90). This process relies on efficient delivery systems (such as mRNA lipid nanoparticles, viral vectors, or dendritic cell loading techniques) to present antigens to immune cells, augmented by adjuvants (such as TLR agonists) to enhance immune activation, thereby improving immune system recognition and killing capacity against tumor cells. Research indicates that while mRNA vaccines have demonstrated efficacy across various malignancies, progress in glioma applications has been comparatively gradual (91).

Currently, the application of personalized mRNA vaccines in GBM treatment is being actively explored (92), providing novel directions for the integration of PDGFRA-targeted vaccines. While mRNA vaccine applications remain in the experimental phase, their preliminarily confirmed immune-activating potential may provide additional theoretical support and practical guidance for PDGFRA vaccine design through in-depth analysis of glioma-specific immune response mechanisms. Future developments hold promise for more targeted and efficacious therapeutic regimens (93).

### PDGFRA vaccine design and optimization

PDGFRA vaccine design necessitates consideration of multiple factors, including personalized antigen selection, multi-antigen targeting, and refinement of vaccine platforms or adjuvants—elements that constitute critical determinants for enhancing therapeutic efficacy in glioma vaccination strategies.

Antigen selection constitutes the core element of vaccine design. PDGFRA mutations or aberrant expression in gliomas can generate immunogenic neoantigens—tumor-specific antigens capable of immune system recognition and induction of specific T cell responses (94–97). Ideal PDGFRA vaccine neoantigens should demonstrate high expression in tumor cells while maintaining specificity for individual subjects or subject subgroups receiving the antigen target, thereby reducing off-target toxicity risks and enhancing immunotherapeutic specificity (98, 99). Implementation of this strategy depends on the integrated application of multi-omics technologies (genomics, transcriptomics, and immunopeptidomics).

The development of high-throughput sequencing technologies has provided powerful tools for neoantigen identification. Comprehensive identification of tumor-specific mutations can be achieved through genomic and transcriptomic sequencing of neoplastic cells (100). Furthermore, bioinformatic analyses can predict the binding capacity of mutation-derived peptides to patients’ major histocompatibility complex (MHC) molecules, evaluating their potential for T cell recognition (101, 102).

Delivery systems play a crucial role in vaccine efficacy, not only protecting antigens from degradation but also effectively delivering them to antigen-presenting cells, thereby efficiently activating immune responses. Delivery systems can influence immune reactions through multiple mechanisms, including enhancement of antigen uptake and cross-presentation, as well as activation of inflammatory signaling pathways. Currently, various delivery systems are under investigation, including viral vectors, liposomes, and polymeric nanoparticles, each possessing unique advantages and limitations.

Among these, nanoparticles have emerged as promising tools in cancer therapy, demonstrating significant research potential in targeted drug and gene delivery. These tools can influence immune cells through various mechanisms, such as facilitating targeted delivery and promoting endosomal escape, thereby enabling release of drugs, antigens, and adjuvants at intended targets (103–105). Surface modification of nanoparticles with targeting ligands can further optimize delivery to lymphoid organs or antigen-presenting cells, while their unique physicochemical properties can enhance dendritic cell recognition and uptake (106). The utilization of nanoparticle systems for delivering immunotherapeutic agents is being actively explored, potentially establishing them as effective options for future PDGFRA tumor vaccine therapies. To further enhance vaccine immunogenicity, the application of adjuvants is essential.

Furthermore, adjuvants—key substances that enhance immune responses—are typically used in conjunction with antigens to augment the durability and intensity of immune reactions (107, 108). Adjuvants function through multiple mechanisms, including prolonging antigen exposure, promoting activation of antigen-presenting cells, and inducing cytokine release, ultimately enhancing adaptive immune responses (109). Concurrently, appropriate adjuvant selection warrants consideration to avoid excessive immune system activation, which may precipitate inflammation or other adverse reactions (110). Commonly employed adjuvants encompass aluminum salts, incomplete Freund’s adjuvant (IFA), Toll-like receptor (TLR) agonists, and cytokines (111, 112). Richard G. Everson et al. investigated the efficacy of TLR agonists combined with autologous tumor lysate-pulsed dendritic cell (ATL-DC) vaccines in malignant glioma patients (NCT01204684), demonstrating safety and enhanced systemic immune responses, evidenced by increased interferon gene expression and alterations in immune cell activation (113).

## Preclinical research, clinical status, and translational challenges

### Preclinical research

PDGFRA plays a pivotal role in glioma pathobiology, with its overexpression and activation representing critical drivers of glioma cell proliferation, angiogenesis, and invasion (114–116). Preclinical investigations have established a robust foundation for elucidating the complex mechanisms through which PDGFRA functions within these neoplasms (**Table 1**).

**Table 1:** Recent preclinical research on glioma vaccines.

Model name	Mouse strain	Vaccine format	Outcomes	Reference
Syngeneic mouse models	C57BL/6 mice	peptide	Increased survival rate	(172)
Syngeneic mouse models	C57BL/6J mice	peptide	Increased survival rate	(173)
Syngeneic mouse models	C57BL/6N mice	peptide	Increased survival rate	(174)
Humanized mouse models	A2.DR1 mice	peptide	Vaccination reduces growth of tumors	(175)
Syngeneic mouse models	C57BL/6 mice	peptide	Increased survival rate	(176)
Syngeneic mouse models	C57BL/6 mice	mRNA	mOS:40 vs 31 days	(177)
Spontaneous glioma	Dog	mRNA	mOS:139 vs 35 days	(92)
Humanized mouse models	NOG mice	Dendritic cell	mOS:60 vs 40 days	(178)
Syngeneic mouse models	C57BL/6 mice	Dendritic cell	Increased survival rate	(179)
Syngeneic mouse models	C57BL/6 mice	Dendritic cell	mOS:38.5 vs 26.8 days	(180)

Hongyan Zou et al. generated malignant glioma murine models via knockin of mutant PDGFRA, based on cell-autonomous activation of PDGFRA in oligodendrocyte precursor cells (OPCs) (117). Another research team constructed high-grade glioma (HGG) murine models through intracranial implantation of p53-deficient primary astrocytes transduced with PDGFRA D842V mutation (118). Investigations have demonstrated that PDGFRA can induce activation of downstream signaling pathways, including PI3K/AKT and RAS/RAF/MAPK cascades, which play crucial roles in cellular growth, survival, and migration (116, 119–121). Recent studies by Soniya Bastola et al. revealed that endothelial-secreted Endocan (ESM1) enhances glioma proliferation, migration, and angiogenic capacity through direct binding and activation of PDGFRA, subsequently activating PI3K and ERK1/2 pathways (42).

## Systematic assessment of current clinical status

### Research progress of current glioma vaccines

Current vaccine research conducted across varying pathological subtypes of gliomas, including glioblastoma, astrocytoma, and diffuse glioma, has yielded a series of breakthrough achievements (**Tables 2**, **3**).

**Table 2:** Clinical trials of PDGFRA in glioma.

Trial	Phases	Vaccine	Vaccine format	Status	Enrollment	Key outcomes	Reference
NCT01130077	I	TetGAA peptide vaccine	Peptide	Completed	60	mPFS:9.9 months	(181)
NCT01222221	I	IMA950	Peptide	Completed	45	PFS-6:74% mOS:15.3 months	(182)
NCT01250470	I	SurVaxM	Peptide	Completed	9	mPFS:17.6 weeks mOS:86.6 weeks	(183)
NCT02454634	I	IDH1-vac	Peptide	Completed	39	ORR:84.4% 3-year PFS:63%	(123)
NCT02709616	I	TAA-based tumor vaccines	Dendritic cell	Completed	10	mOS:19 months	(184)
NCT02149225	I	APVAC	DNA/RNA	Completed	16	mPFS:14.2 months mOS:29 months	(185)
NCT00293423	I/II	HSPPC-96	Peptide	Completed	41	mOS:42.6 weeks	(128)
NCT01291420	I/II	WT1-mRNA/DC	Dendritic cell	Completed	48	mOS:43.7 months	(186)
NCT03400917	II	AV-GBM-1	Dendritic cell	Unknown	55	mPFS:10.4 months mOS:16.0 months	(187)
NCT00045968	III	DCVax-L	Dendritic cell	Unknown	331	nGBM:mOS 19.3 months rGBM:mOS 13.2months	(130)
NCT02455557	II	SurVaxM	Peptide	Active	66	mPFS:11.4 months mOS:25.9 months	(122)

**Table 3:** Identified and predicted PDGFRA epitopes for glioma immunotherapy.

Antigen source	Specific peptide/Variation	HLA restriction	Discovery method	Evidence
Wild-type PDGFRA	IMA950 (Multi-peptide cocktail)	HLA-A*02	Ligandome analysis (Mass Spec)	Included in the IMA950 vaccine; naturally presented on GBM surface in patients with PDGFRA overexpression (Schoor et al., Cancer Res).
Wild-type PDGFRA	KTS (and other APVAC1 peptides)	Class I (A02, A24)	GAPVAC Trial (Immunopeptidomics)	Personalized vaccine targets identified directly from patient tumor tissue; PDGFRA peptides were among the frequent “warehouse” targets selected (Hilf et al., Nature 2019).
Mutant PDGFRA	D842V (Exon 18)	HLA-A*02 (Predicted)	In silico NetMHC prediction	The most common activating mutation in GBM; generates a high-affinity neoepitope distinct from wild-type, minimizing autoimmunity risk (Pre-clinical rationale).
Fusion/Del	PDGFRA-Δ8/9 (Deletion)	Patient-specific	Transcriptome Sequencing	In-frame deletions (e.g., Gas7-PDGFRA or exon deletions) create novel junctional epitopes suitable for personalized mRNA or peptide vaccines (Ozawa et al., Genes Dev).

Among these, peptide vaccines represent a classical type in glioma vaccination. In a phase II clinical trial of the Survivin-targeted vaccine SurVaxM (NCT02455557), GBM patients who failed the Stupp protocol achieved a median overall survival (mOS) of 25.9 months when combined with temozolomide adjuvant therapy, with both methylated and unmethylated GBM patients demonstrating significant clinical benefit (122). Michael Platten’s team conducted a multicenter phase I clinical study (NCT02454634) of an IDH1(R132H)-targeted peptide vaccine, demonstrating that specific peptide vaccination (IDH1-vac) for grade III-IV (WHO classification) gliomas yielded 3-year progression-free and mortality-free rates of 63% and 84%, respectively (123). Rindopepimut (CDX-110), a peptide vaccine targeting the EGFRvIII mutation, showed survival benefits in several phase II clinical trials for GBM patients (124–126), yet a subsequent phase III trial (NCT01480479) combining CDX-110 with chemotherapy failed to demonstrate significant clinical benefit (mOS 20.1 months vs. 20.0 months) (127).

Given GBM’s pronounced heterogeneity, vaccines targeting standardized single antigens may fail to produce durable antitumor effects. Consequently, multi-antigen vaccines targeting multiple tumor antigens and personalized vaccines theoretically achieve more effective tumor cell elimination. A phase II single-arm trial by Andrew T. Parsa’s research team evaluated the HSPPC-96 vaccine’s safety and efficacy in recurrent GBM (rGBM) patients, demonstrating an mOS of 42.6 weeks (128); their further research indicated this vaccine improved survival rates in newly diagnosed GBM (nGBM) patients receiving standard of care (SOC), with an mOS of 23.8 months (129). The dendritic cell vaccine DCVax-L has shown significant clinical benefit in glioblastoma treatment, with a phase III clinical trial (NCT00045968) demonstrating that, compared to external controls, DCVax-L treatment extended mOS in both nGBM patients (19.3 vs. 16.5 months) and rGBM patients (13.2 vs. 7.8 months) (130).

The research landscape concerning PDGFRA-driven immune evasion in gliomas has yielded significant successes, primarily rooted in the comprehensive characterization of PDGFRA’s oncogenic role. Preclinical models have unequivocally demonstrated PDGFRA’s pivotal involvement in fueling glioma proliferation, angiogenesis, and invasion, further elucidated through the identification of key downstream signaling pathways, including PI3K/AKT and RAS/RAF/MAPK, which are critical for tumor survival and migration. The discovery of Endocan’s direct interaction and activation of PDGFRA offers a novel target for enhancing glioma progression and angiogenic capacity. These foundational discoveries have spurred the development of diverse glioma vaccine platforms, ranging from peptide and tumor-associated antigen vaccines to DNA/RNA and dendritic cell vaccines, many of which have progressed into clinical trials across various stages of glioma. Encouragingly, some of these vaccine strategies have shown preliminary clinical efficacy, with certain cohorts demonstrating potential for extended overall survival when used in combination therapies or in specific patient populations. However, the field is also marked by considerable failures and limitations. The pivotal phase III trial failure of the EGFRvIII-targeted peptide vaccine Rindopepimut (CDX-110) serves as a stark reminder of the translational chasm. Furthermore, the profound heterogeneity inherent in glioblastoma necessitates multifaceted approaches, as single-antigen targeting has proven insufficient for eliciting durable anti-tumor immune responses, and issues of inadequate immunogenicity remain a concern. The ultimate clinical validation of other promising vaccine candidates, such as DCVax-L and HSPPC-96, is still pending, underscoring the challenges in achieving consistent and robust clinical benefits.

### Clinical application of existing PDGFRA-targeted drugs

PDGFRA has garnered substantial attention from researchers as a potential therapeutic target in oncology. Various PDGFRA-targeted therapeutic strategies have been explored, including tyrosine kinase inhibitors and monoclonal antibodies (45, 131, 132). The complexity of PDGFRA signaling and its crosstalk with other receptor tyrosine kinases may contribute to resistance against PDGFRA inhibitors, reflecting limited efficacy across multiple clinical trials. Current research indicates that previous tyrosine kinase inhibitors targeting PDGFRA (such as dasatinib and imatinib) demonstrated limited efficacy in clinical trials, potentially due to poor drug tolerance and ineffective blood-brain barrier penetration (133, 134). In contrast, avapritinib has shown preliminary efficacy in HGG patients. Recent studies have preliminarily confirmed avapritinib’s significant potential clinical value in treating pediatric HGG patients carrying specific PDGFRA gene variants (particularly amplification or exon 18 mutations), though validation with additional clinical data is necessary due to small sample sizes. A phase I/II clinical trial (NCT04773782) is currently underway to further evaluate its safety and efficacy (45).

Furthermore, preclinical studies have investigated strategies combining PDGFRA inhibitors with other therapies such as radiotherapy, demonstrating synergistic antitumor effects in certain glioma subtypes (58). Consequently, enhanced understanding of PDGFRA’s role and mechanisms in gliomas could facilitate the development of PDGFRA-related vaccine therapeutic strategies, thereby improving treatment outcomes for patients with this devastating disease.

### Bottlenecks in clinical translation

Although avapritinib and other novel tyrosine kinase inhibitors (TKIs) have shown potential in patients with specific PDGFRA mutations, TKI drugs generally face the problems of drug resistance and limited blood-brain barrier penetration efficiency (135). This highlights the necessity of developing therapies with different mechanisms of action, such as therapeutic vaccines. By mobilizing the systemic immune system, vaccines have the potential to generate more durable memory responses and may form synergistic effects with targeted drugs like TKIs.

Translational barriers impeding PDGFRA-targeted vaccines’ progression from research prospects to clinical success encompass multiple practical and technical challenges, including not only the immunosuppressive tumor microenvironment and blood-brain barrier impediments discussed in previous sections, but also complex resistance mechanisms and challenges in real-world technology standardization (136–140).

Glioma cells can develop resistance through multiple pathways, including promotion of epigenetic regulation, activation of bypass signaling pathways, and enhancement of DNA repair capabilities. GBM cells typically exhibit resistance to temozolomide (TMZ), with mechanisms involving O6-methylguanine-DNA methyltransferase (MGMT) expression, mismatch repair gene mutations, and interactions between m6A and histone modifications (141). Additionally, glioma cells can induce resistance through epigenetic modifications, remodeling of the tumor microenvironment, and acquisition of stem cell-like characteristics (137). Activation of multiple signaling pathways also provides opportunities for resistance development. PI3K-AKT, NOTCH, WNT/β-catenin, and MAPK signaling pathways commonly exhibit abnormal activation in gliomas, which not only promotes tumor growth and invasion but also leads to resistance against existing therapeutic approaches (142–145). To overcome this challenge, researchers have explored various synergistic treatment modalities, with combined targeting of multiple signaling pathways considered an effective strategy. Studies have found that EGFR and PDGFRA are frequently co-expressed in gliomas, with EGFR inhibitors affecting PDGFRA signaling through functional transactivation (19). Combined inhibition of EGFR and PI3K/AKT/mTOR pathways can enhance antitumor activity and effectively delay resistance development (146).

Upregulation of immune checkpoint molecules in glioma cells following vaccine therapy may constitute one reason for vaccine failure (147, 148). To counter such immunosuppressive mechanisms, immune checkpoint inhibitors (ICIs) represent ideal adjuncts to vaccine therapy, aiming to overcome potential immunosuppressive or immunologically “cold” tumor microenvironments through inhibition of negative co-stimulatory molecules (such as PD-1/PD-L1) (149, 150). Such combination therapies have demonstrated promising clinical outcomes in other malignancies (including non-small cell lung cancer, melanoma, hepatocellular carcinoma, and bladder cancer), with confirmation in gliomas pending continued investigation (151–153). Preclinical studies have confirmed that PD-1 blockade enhances vaccine-induced immune responses in orthotopic mouse GBM models (149). Additional clinical trials investigating ICI and vaccine combinations for glioma treatment are currently underway (NCT02287428, NCT02960230, NCT04201873).

Glioma heterogeneity imposes stringent requirements for precise antigen presentation and efficient drug delivery, constituting a key technical challenge in tumor vaccine development. Currently, neoantigen vaccines tailored to patient-specific mutations have demonstrated potential to induce effective antitumor immune responses across multiple cancer types (152, 154–157). Concurrently, researchers have developed immune infiltration-related risk models that may facilitate selection of patients suitable for immunotherapy through gene expression analysis, promoting individualized immunotherapeutic approaches for improved clinical outcomes (158). Considering each patient’s tumor’s unique genetic and immunological characteristics, such personalized medical approaches may be crucial for realizing PDGFRA vaccine potential, maximizing therapeutic efficacy while minimizing vaccine toxicity.

Various modern emerging technologies offer new tools for vaccine development and combination strategies. Researchers have developed nanomedicines capable of traversing the BBB, reactivating immunosuppressive TME and triggering anti-tumor immune cascades by activating interferon gene pathways and inhibiting the PD-1/PD-L1 signaling axis (59). Oncolytic viruses, as a novel immunotherapy, exert direct oncolytic effects and enhance antigen exposure, converting “cold” TME to “hot” TME in gliomas, ultimately activating anti-tumor immunity. A phase II clinical trial demonstrated G47Δ (oncolytic herpes virus) efficacy and safety for residual or recurrent glioblastoma, with an 84.2% one-year survival rate and median OS and PFS of 20.2 and 4.7 months, respectively. Additionally, PVSRIPO (recombinant non-pathogenic poliovirus-rhinovirus chimera) demonstrated efficacy in a phase I clinical trial enrolling 61 glioma patients (NCT01491893) (159).

From a clinical translation perspective, PDGFRA-targeted vaccine strategies for glioma offer a compelling duality of significant promise and formidable challenges. The inherent capacity of immunotherapies, particularly vaccines, to overcome glioma’s pronounced cellular and molecular heterogeneity through multi-antigenic or personalized approaches presents a distinct advantage, potentially eliciting more sustained and robust anti-tumor immune responses than conventional monotherapies. This approach also holds the promise of establishing durable immunological memory, crucial for long-term disease control. Furthermore, the synergistic potential of integrating vaccine therapies with existing treatments like chemotherapy and emerging immune checkpoint inhibitors offers a powerful avenue to augment overall therapeutic efficacy. Notably, many vaccine candidates have demonstrated favorable safety profiles in early-stage investigations, a critical factor for patient acceptance and broader clinical adoption.

However, the path to widespread clinical translation is impeded by substantial obstacles. The blood-brain barrier (BBB) remains a significant physical impediment, limiting the efficient delivery and *in situ* activation of vaccine components within the tumor microenvironment. This challenge is exacerbated by the profoundly immunosuppressive nature of the glioma milieu, which actively impairs anti-tumor immune cell function and persistence, presenting a critical hurdle for ensuring effective antigen presentation and robust cytotoxic T lymphocyte activation. The development and standardization of clinical-grade manufacturing processes, particularly for personalized vaccines, introduce substantial logistical and cost barriers, demanding scalable and reproducible technologies. Moreover, the observed discrepancies between promising preclinical outcomes and clinical trial results underscore the limitations of current animal models, which often fail to fully recapitulate the “cold tumor” characteristics of human gliomas, hindering accurate prediction of *in vivo* efficacy (160). Consequently, advancing PDGFRA vaccines from bench to bedside necessitates rigorous preclinical evaluation of safety and immunogenicity, alongside optimization of administration methods, dosages, and scheduling, all while navigating complex regulatory landscapes and refining patient selection biomarkers to identify individuals most likely to benefit from these innovative immunotherapeutic interventions.

## Future development directions and research priorities

The treatment paradigm for gliomas presents a formidable clinical challenge. Tumor vaccines constitute an effective approach for enhancing therapeutic outcomes in glioma patients; however, compared with other solid tumors, barriers such as cranial biological barriers and the unique tumor and immune microenvironment impact vaccine efficacy. Consequently, researchers have conducted extensive investigations in immunotherapy and precision oncology domains to explore novel methodologies. Currently, select glioma vaccines have demonstrated positive interim clinical research outcomes, though developmental potential remains substantial.

### Personalized vaccine strategies based on neoantigens

Glioma PDGFRA vaccines necessitate thorough consideration of tumor immune complexity and inter-patient heterogeneity. Tumor heterogeneity, immune status, and genetic background may influence vaccine efficacy and constrain immune cell infiltration and function. Consequently, personalized vaccine design is progressively emerging as a future developmental trend. Through genomic and immunomic analyses of patient tumors, customized therapeutic strategies adapted to individual tumor characteristics can be provided, acknowledging each patient’s unique tumor biology, thereby substantially enhancing therapeutic outcomes (161, 162). Future development of personalized vaccines targeting patient-specific neoantigens, through genomic identification of mutations and peptide vaccine design, aims to achieve superior tumor regression and anti-tumor responses (163). Additionally, optimization of vaccine administration protocols, including dose selection, delivery route refinement, and rational timing arrangements, constitutes an important approach for improving vaccine efficacy.

### Utilizing advanced technologies to optimize vaccine design and delivery

Novel technological developments provide powerful tools for in-depth investigation of glioma vaccines. With advancements in bioinformatics and artificial intelligence, challenges in identifying and targeting novel antigens, including PDGFRA-related antigens, may potentially be resolved (164). Multi-omics technologies comprehensively elucidate molecular characteristics of tumors and immune systems. Through utilization of genomics, transcriptomics, proteomics, and metabolomics, potential vaccine targets can be identified, immune profiles of tumor microenvironments characterized, and vaccine influences on immune cell activity elucidated (165). Furthermore, identification of biomarkers predicting vaccine response, such as PDGFRA status or immune infiltration characteristics, facilitates selection of patients most likely to benefit from vaccine therapy. Development of novel delivery systems constitutes another priority direction. Systems based on nanoparticles, viral vectors, or liposomes capable of specifically targeting glioma cells or tumor microenvironments enhance vaccine capacity to traverse the BBB while reducing off-target effects. Additionally, exploration of ultrasound or other physical techniques to temporarily disrupt the BBB represents a potentially promising strategy for promoting vaccine delivery.

### Combined treatment strategies to enhance tumor vaccine efficacy

**Figure 4:**
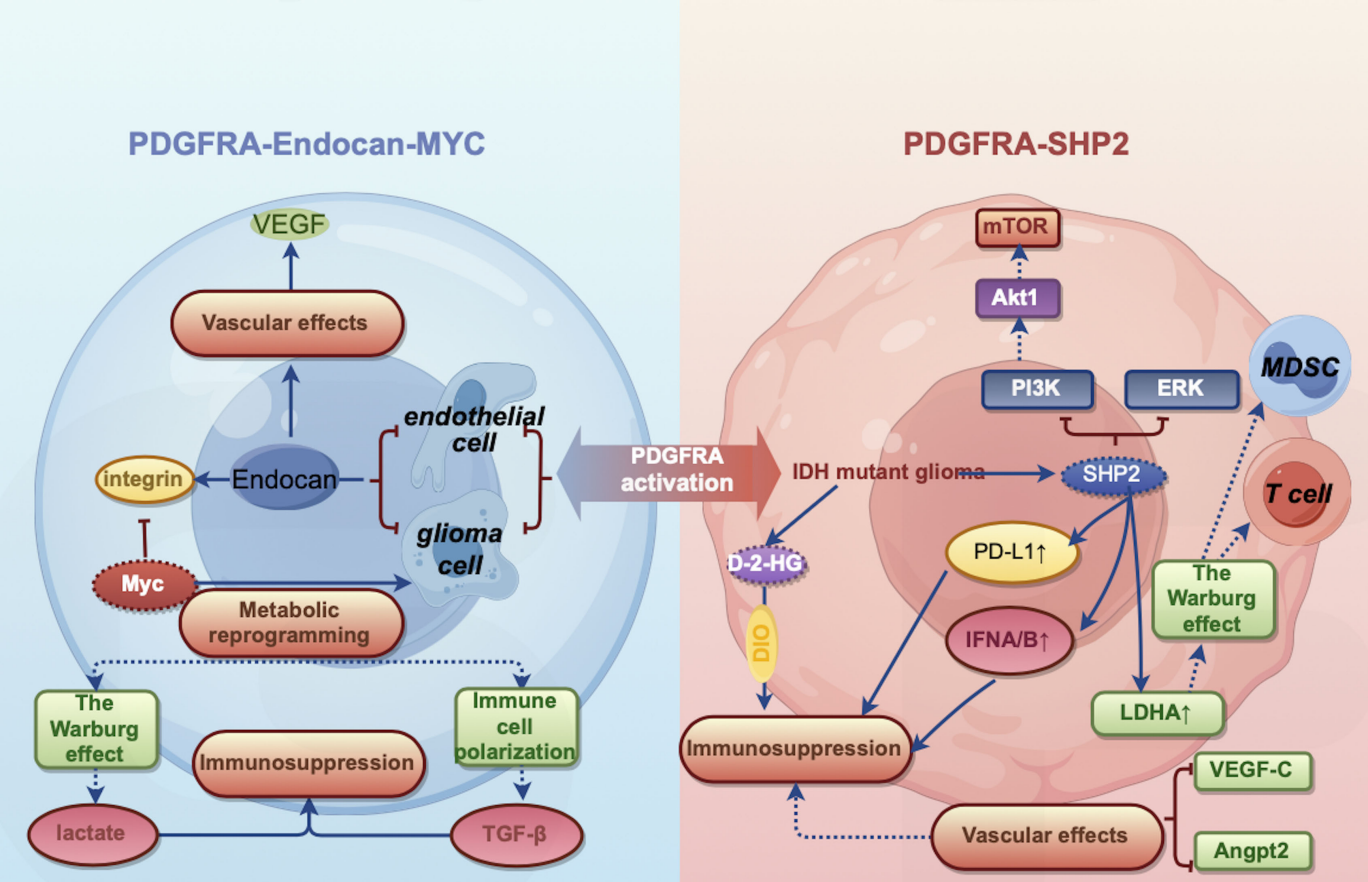
PDGFRA-targeted vaccine strategy in glioma immunotherapy: Mechanism, challenges, and future directions. The illustration depicts the design and mechanism of PDGFRA-targeting vaccines (e.g., peptide, mRNA, DC, or viral vector platforms), which utilize antigens such as full-length PDGFRA, extracellular domains, or mutation-specific epitopes to activate APCs and induce cytotoxic (CD8+) and helper (CD4+) T-cell responses, ultimately leading to tumor cell killing and potential remodeling of the immunosuppressive TME. Key clinical challenges include the blood-brain barrier, tumor heterogeneity, immune-suppressive TME, and limited immunogenicity of PDGFRA as a self-antigen. Future optimization may involve combinatorial approaches (e.g., immune checkpoint inhibitors, chemotherapy), nano-delivery systems to bypass the BBB, personalized neoantigen strategies, and TME-modulating therapies to enhance efficacy.

Immunotherapy resistance constitutes a critical bottleneck constraining glioma vaccine efficacy enhancement, with in-depth analysis of underlying mechanisms and exploration of effective countermeasures representing an important future research direction. Tumor microenvironment complexity significantly influences vaccine efficacy, with abundant immunosuppressive cells and aberrant immune checkpoint activation substantially limiting immune cell infiltration and cytotoxic function (166, 167). Addressing this challenge, combined treatment strategies emerge as crucial breakthrough approaches. Concurrent administration of glioma tumor vaccines with immune checkpoint inhibitors (such as PD-1/PD-L1 inhibitors) effectively blocks immunosuppressive signaling pathways, reactivating T-cell anti-tumor activity and enhancing vaccine-induced immune responses. Simultaneously, PDGFRA-targeted therapies combined with vaccines precisely modulate critical tumor cell signaling pathways, overcoming resistance arising from signaling abnormalities. Designing vaccines targeting multiple antigens constitutes another important strategy preventing immune evasion due to antigen loss. Multi-stage combined therapeutic approaches may provide promising supplementary regimens, with PDGFRA tumor vaccines administered concurrently with chemotherapy, radiotherapy, or immune checkpoint inhibitors potentially further enhancing therapeutic outcomes, overcoming tumor resistance, and achieving more sustained tumor control (168–171) (**Figure 4**).

## Conclusion

PDGFRA-mediated signaling networks establish complex interactions between glioma cells and the immunosuppressive microenvironment, constituting a core barrier to immunotherapeutic efficacy. Advances in multi-omics technologies and neoantigen identification are facilitating development of highly specific, individualized PDGFRA-targeted vaccine designs. Combined with next-generation delivery systems and effective adjuvants, such vaccines may induce robust and durable anti-tumor immune responses, potentially facilitating transformation of gliomas from “immunologically cold” to “hot” tumors. Prospectively, deep integration of artificial intelligence, molecular typing, nanotechnology, and immune engineering will play critical roles in overcoming glioma immune evasion and achieving precise immunotherapy, offering new therapeutic prospects for patients.

## References

[1] Weller M Wen PY Chang SM Dirven L Lim M Monje M . Glioma. Nat Rev Dis Primers. (2024) 10:33. doi: 10.1038/s41572-024-00516-y, PMID: 38724526

[2] Sharma P Aaroe A Liang J Puduvalli VK . Tumor microenvironment in glioblastoma: Current and emerging concepts. Neurooncol Adv. (2023) 5:vdad009. doi: 10.1093/noajnl/vdad009, PMID: 36968288 PMC10034917

[3] Yabo YA Niclou SP Golebiewska A . Cancer cell heterogeneity and plasticity: A paradigm shift in glioblastoma. Neuro Oncol. (2022) 24:669–82. doi: 10.1093/neuonc/noab269, PMID: 34932099 PMC9071273

[4] Paugh BS Zhu X Qu C Endersby R Diaz AK Zhang J . Novel oncogenic PDGFRA mutations in pediatric high-grade gliomas. Cancer Res. (2013) 73:6219–29. doi: 10.1158/0008-5472.Can-13-1491, PMID: 23970477 PMC3800209

[5] Rogers MA Fantauzzo KA . The emerging complexity of PDGFRs: activation, internalization and signal attenuation. Biochem Soc Trans. (2020) 48:1167–76. doi: 10.1042/bst20200004, PMID: 32369556 PMC7722362

[6] Jackson CM Choi J Lim M . Mechanisms of immunotherapy resistance: lessons from glioblastoma. Nat Immunol. (2019) 20:1100–9. doi: 10.1038/s41590-019-0433-y, PMID: 31358997

[7] Adhikaree J Moreno-Vicente J Kaur AP Jackson AM Patel PM . Resistance mechanisms and barriers to successful immunotherapy for treating glioblastoma. Cells. (2020) 9:263. doi: 10.3390/cells9020263, PMID: 31973059 PMC7072315

[8] Akiyama T Yasuda T Uchihara T Yasuda-Yoshihara N Tan BJY Yonemura A . Stromal reprogramming through dual PDGFRα/β Blockade boosts the efficacy of anti-PD-1 immunotherapy in fibrotic tumors. Cancer Res. (2023) 83:753–70. doi: 10.1158/0008-5472.Can-22-1890, PMID: 36543251

[9] Yang D Duan MH Yuan QE Li ZL Luo CH Cui LY . Suppressive stroma-immune prognostic signature impedes immunotherapy in ovarian cancer and can be reversed by PDGFRB inhibitors. J Transl Med. (2023) 21:586. doi: 10.1186/s12967-023-04422-x, PMID: 37658364 PMC10472577

[10] Cenciarelli C Marei HE Zonfrillo M Pierimarchi P Paldino E Casalbore P . PDGF receptor alpha inhibition induces apoptosis in glioblastoma cancer stem cells refractory to anti-Notch and anti-EGFR treatment. Mol Cancer. (2014) 13:247. doi: 10.1186/1476-4598-13-247, PMID: 25380967 PMC4235989

[11] Yan D Kowal J Akkari L Schuhmacher AJ Huse JT West BL . Inhibition of colony stimulating factor-1 receptor abrogates microenvironment-mediated therapeutic resistance in gliomas. Oncogene. (2017) 36:6049–58. doi: 10.1038/onc.2017.261, PMID: 28759044 PMC5666319

[12] Guérit E Arts F Dachy G Boulouadnine B Demoulin JB . PDGF receptor mutations in human diseases. Cell Mol Life Sci. (2021) 78:3867–81. doi: 10.1007/s00018-020-03753-y, PMID: 33449152 PMC11072557

[13] Funa K Sasahara M . The roles of PDGF in development and during neurogenesis in the normal and diseased nervous system. J Neuroimmune Pharmacol. (2014) 9:168–81. doi: 10.1007/s11481-013-9479-z, PMID: 23771592 PMC3955130

[14] Chojnacki A Mak G Weiss S . PDGFRα expression distinguishes GFAP-expressing neural stem cells from PDGF-responsive neural precursors in the adult periventricular area. J Neurosci. (2011) 31:9503–12. doi: 10.1523/jneurosci.1531-11.2011, PMID: 21715615 PMC6623177

[15] Lee K Park SO Choi PC Ryoo SB Lee H Peri LE . Molecular and functional characterization of detrusor PDGFRα positive cells in spinal cord injury-induced detrusor overactivity. Sci Rep. (2021) 11:16268. doi: 10.1038/s41598-021-95781-2, PMID: 34381120 PMC8357952

[16] Goenka A Song X Tiek D Iglesia RP Lu M Zeng C . Oncogenic long noncoding RNA LINC02283 enhances PDGF receptor A-mediated signaling and drives glioblastoma tumorigenesis. Neuro Oncol. (2023) 25:1592–604. doi: 10.1093/neuonc/noad065, PMID: 36988488 PMC10479875

[17] Hamada T Akahane T Yokoyama S Higa N Kirishima M Matsuo K . An oncogenic splice variant of PDGFRα in adult glioblastoma as a therapeutic target for selective CDK4/6 inhibitors. Sci Rep. (2022) 12:1275. doi: 10.1038/s41598-022-05391-9, PMID: 35075231 PMC8786844

[18] Rogers MA Campaña MB Long R Fantauzzo KA . PDGFR dimer-specific activation, trafficking and downstream signaling dynamics. J Cell Sci. (2022) 135:jcs259686. doi: 10.1242/jcs.259686, PMID: 35946433 PMC9482349

[19] Chakravarty D Pedraza AM Cotari J Liu AH Punko D Kokroo A . EGFR and PDGFRA co-expression and heterodimerization in glioblastoma tumor sphere lines. Sci Rep. (2017) 7:9043. doi: 10.1038/s41598-017-08940-9, PMID: 28831081 PMC5567352

[20] Piccaluga PP Cascianelli C Inghirami G . Tyrosine kinases in nodal peripheral T-cell lymphomas. Front Oncol. (2023) 13:1099943. doi: 10.3389/fonc.2023.1099943, PMID: 36845713 PMC9946040

[21] Popielarczyk TL Huckle WR Barrett JG . Human bone marrow-derived mesenchymal stem cells home via the PI3K-akt, MAPK, and jak/stat signaling pathways in response to platelet-derived growth factor. Stem Cells Dev. (2019) 28:1191–202. doi: 10.1089/scd.2019.0003, PMID: 31190615

[22] Zhou LL Xu XY Ni J Zhao X Zhou JW Feng JF . T-cell lymphomas associated gene expression signature: Bioinformatics analysis based on gene expression Omnibus. Eur J Haematol. (2018) 100:575–83. doi: 10.1111/ejh.13051, PMID: 29505095

[23] Jiang H Liao J Wang L Jin C Mo J Xiang S . The multikinase inhibitor axitinib in the treatment of advanced hepatocellular carcinoma: the current clinical applications and the molecular mechanisms. Front Immunol. (2023) 14:1163967. doi: 10.3389/fimmu.2023.1163967, PMID: 37325670 PMC10264605

[24] Dutta S Ganguly A Chatterjee K Spada S Mukherjee S . Targets of immune escape mechanisms in cancer: basis for development and evolution of cancer immune checkpoint inhibitors. Biol (Basel). (2023) 12:218. doi: 10.3390/biology12020218, PMID: 36829496 PMC9952779

[25] Baskin Y Kocal GC Kucukzeybek BB Akbarpour M Kayacik N Sagol O . PDGFRA and KIT mutation status and its association with clinicopathological properties, including DOG1. Oncol Res. (2016) 24:41–53. doi: 10.3727/096504016x14576297492418, PMID: 27178821 PMC7838738

[26] Phillips JJ Aranda D Ellison DW Judkins AR Croul SE Brat DJ . PDGFRA amplification is common in pediatric and adult high-grade astrocytomas and identifies a poor prognostic group in IDH1 mutant glioblastoma. Brain Pathol. (2013) 23:565–73. doi: 10.1111/bpa.12043, PMID: 23438035 PMC3715570

[27] Coy S Wang S Stopka SA Lin JR Yapp C Ritch CC . Single cell spatial analysis reveals the topology of immunomodulatory purinergic signaling in glioblastoma. Nat Commun. (2022) 13:4814. doi: 10.1038/s41467-022-32430-w, PMID: 35973991 PMC9381513

[28] Li Y Shen X Zhang J Xian X Chen S Zeng J . Adult diffuse IDH-wildtype lower-grade gliomas with PDGFRA gain/amplification should be upgraded as glioblastoma. J Neuropathol Exp Neurol. (2025) 84:634–41. doi: 10.1093/jnen/nlaf039, PMID: 40238212

[29] Indio V Astolfi A Tarantino G Urbini M Patterson J Nannini M . Integrated molecular characterization of gastrointestinal stromal tumors (GIST) harboring the rare D842V mutation in PDGFRA gene. Int J Mol Sci. (2018) 19:732. doi: 10.3390/ijms19030732, PMID: 29510530 PMC5877593

[30] Ozawa T Brennan CW Wang L Squatrito M Sasayama T Nakada M . PDGFRA gene rearrangements are frequent genetic events in PDGFRA-amplified glioblastomas. Genes Dev. (2010) 24:2205–18. doi: 10.1101/gad.1972310, PMID: 20889717 PMC2947772

[31] Mbah NE Myers AL Sajjakulnukit P Chung C Thompson JK Hong HS . Therapeutic targeting of differentiation-state dependent metabolic vulnerabilities in diffuse midline glioma. Nat Commun. (2024) 15:8983. doi: 10.1038/s41467-024-52973-4, PMID: 39419964 PMC11487135

[32] Wu M Wang T Ji N Lu T Yuan R Wu L . Multi-omics and pharmacological characterization of patient-derived glioma cell lines. Nat Commun. (2024) 15:6740. doi: 10.1038/s41467-024-51214-y, PMID: 39112531 PMC11306361

[33] Martinho O Longatto-Filho A Lambros MB Martins A Pinheiro C Silva A . Expression, mutation and copy number analysis of platelet-derived growth factor receptor A (PDGFRA) and its ligand PDGFA in gliomas. Br J Cancer. (2009) 101:973–82. doi: 10.1038/sj.bjc.6605225, PMID: 19707201 PMC2743351

[34] Rautajoki KJ Jaatinen S Hartewig A Tiihonen AM Annala M Salonen I . Genomic characterization of IDH-mutant astrocytoma progression to grade 4 in the treatment setting. Acta Neuropathol Commun. (2023) 11:176. doi: 10.1186/s40478-023-01669-9, PMID: 37932833 PMC10629206

[35] Ravi VM Neidert N Will P Joseph K Maier JP Kückelhaus J . T-cell dysfunction in the glioblastoma microenvironment is mediated by myeloid cells releasing interleukin-10. Nat Commun. (2022) 13:925. doi: 10.1038/s41467-022-28523-1, PMID: 35177622 PMC8854421

[36] Magar AG Morya VK Kwak MK Oh JU Noh KC . A molecular perspective on HIF-1α and angiogenic stimulator networks and their role in solid tumors: an update. Int J Mol Sci. (2024) 25:3313. doi: 10.3390/ijms25063313, PMID: 38542288 PMC10970012

[37] Verhaak RG Hoadley KA Purdom E Wang V Qi Y Wilkerson MD . Integrated genomic analysis identifies clinically relevant subtypes of glioblastoma characterized by abnormalities in PDGFRA, IDH1, EGFR, and NF1. Cancer Cell. (2010) 17:98–110. doi: 10.1016/j.ccr.2009.12.020, PMID: 20129251 PMC2818769

[38] Patel AP Tirosh I Trombetta JJ Shalek AK Gillespie SM Wakimoto H . Single-cell RNA-seq highlights intratumoral heterogeneity in primary glioblastoma. Science. (2014) 344:1396–401. doi: 10.1126/science.1254257, PMID: 24925914 PMC4123637

[39] Di Carlo SE Raffenne J Varet H Ode A Granados DC Stein M . Depletion of slow-cycling PDGFRα(+)ADAM12(+) mesenchymal cells promotes antitumor immunity by restricting macrophage efferocytosis. Nat Immunol. (2023) 24:1867–78. doi: 10.1038/s41590-023-01642-7, PMID: 37798557 PMC10602852

[40] Liu X Yu J Li Y Shi H Jiao X Liu X . Deciphering the tumor immune microenvironment of imatinib-resistance in advanced gastrointestinal stromal tumors at single-cell resolution. Cell Death Dis. (2024) 15:190. doi: 10.1038/s41419-024-06571-3, PMID: 38443340 PMC10914684

[41] Cantanhede IG de Oliveira JRM . PDGF family expression in glioblastoma multiforme: data compilation from ivy glioblastoma atlas project database. Sci Rep. (2017) 7:15271. doi: 10.1038/s41598-017-15045-w, PMID: 29127351 PMC5681588

[42] Bastola S Pavlyukov MS Sharma N Ghochani Y Nakano MA Muthukrishnan SD . Endothelial-secreted Endocan activates PDGFRA and regulates vascularity and spatial phenotype in glioblastoma. Nat Commun. (2025) 16:471. doi: 10.1038/s41467-024-55487-1, PMID: 39773984 PMC11707362

[43] Peng G Wang Y Ge P Bailey C Zhang P Zhang D . The HIF1α-PDGFD-PDGFRα axis controls glioblastoma growth at normoxia/mild-hypoxia and confers sensitivity to targeted therapy by echinomycin. J Exp Clin Cancer Res. (2021) 40:278. doi: 10.1186/s13046-021-02082-7, PMID: 34470658 PMC8411541

[44] McLendon R Friedman A Bigner D Van Meir EG Brat DJ Mastrogianakis GM . Comprehensive genomic characterization defines human glioblastoma genes and core pathways. Nature. (2008) 455:1061–8. doi: 10.1038/nature07385, PMID: 18772890 PMC2671642

[45] Mayr L Neyazi S Schwark K Trissal M Beck A Labelle J . Effective targeting of PDGFRA-altered high-grade glioma with avapritinib. Cancer Cell. (2025) 43:740–756.e748. doi: 10.1016/j.ccell.2025.02.018, PMID: 40086436 PMC12121847

[46] Rocha SF Schiller M Jing D Li H Butz S Vestweber D . Esm1 modulates endothelial tip cell behavior and vascular permeability by enhancing VEGF bioavailability. Circ Res. (2014) 115:581–90. doi: 10.1161/circresaha.115.304718, PMID: 25057127

[47] Ahir BK Engelhard HH Lakka SS . Tumor development and angiogenesis in adult brain tumor: glioblastoma. Mol Neurobiol. (2020) 57:2461–78. doi: 10.1007/s12035-020-01892-8, PMID: 32152825 PMC7170819

[48] Ge Z Zhang Q Lin W Jiang X Zhang Y . The role of angiogenic growth factors in the immune microenvironment of glioma. Front Oncol. (2023) 13:1254694. doi: 10.3389/fonc.2023.1254694, PMID: 37790751 PMC10542410

[49] Lu KV Bergers G . Mechanisms of evasive resistance to anti-VEGF therapy in glioblastoma. CNS Oncol. (2013) 2:49–65. doi: 10.2217/cns.12.36, PMID: 23750318 PMC3673744

[50] Penco-Campillo M Pages G Martial S . Angiogenesis and lymphangiogenesis in medulloblastoma development. Biol (Basel). (2023) 12:1028. doi: 10.3390/biology12071028, PMID: 37508458 PMC10376362

[51] Won WJ Deshane JS Leavenworth JW Oliva CR Griguer CE . Metabolic and functional reprogramming of myeloid-derived suppressor cells and their therapeutic control in glioblastoma. Cell Stress. (2019) 3:47–65. doi: 10.15698/cst2019.02.176, PMID: 31225500 PMC6551710

[52] Miklja Z Yadav VN Cartaxo RT Siada R Thomas CC Cummings JR . Everolimus improves the efficacy of dasatinib in PDGFRα-driven glioma. J Clin Invest. (2020) 130:5313–25. doi: 10.1172/jci133310, PMID: 32603316 PMC7524471

[53] Gao J Liang Y Wang L . Shaping polarization of tumor-associated macrophages in cancer immunotherapy. Front Immunol. (2022) 13:888713. doi: 10.3389/fimmu.2022.888713, PMID: 35844605 PMC9280632

[54] Zou X Tang XY Qu ZY Sun ZW Ji CF Li YJ . Targeting the PDGF/PDGFR signaling pathway for cancer therapy: A review. Int J Biol Macromol. (2022) 202:539–57. doi: 10.1016/j.ijbiomac.2022.01.113, PMID: 35074329

[55] Gai QJ Fu Z He J Mao M Yao XX Qin Y . EPHA2 mediates PDGFA activity and functions together with PDGFRA as prognostic marker and therapeutic target in glioblastoma. Signal Transduct Target Ther. (2022) 7:33. doi: 10.1038/s41392-021-00855-2, PMID: 35105853 PMC8807725

[56] Das F Ghosh-Choudhury N Venkatesan B Kasinath BS Ghosh Choudhury G . PDGF receptor-β uses Akt/mTORC1 signaling node to promote high glucose-induced renal proximal tubular cell collagen I (α2) expression. Am J Physiol Renal Physiol. (2017) 313:F291–f307. doi: 10.1152/ajprenal.00666.2016, PMID: 28424212 PMC5582895

[57] Fontana F Giannitti G Marchesi S Limonta P . The PI3K/akt pathway and glucose metabolism: A dangerous liaison in cancer. Int J Biol Sci. (2024) 20:3113–25. doi: 10.7150/ijbs.89942, PMID: 38904014 PMC11186371

[58] Yu X Song X Tiek D Wu R Walker M Horbinski C . Targeting PDGFRA-SHP2 signaling enhances radiotherapy in IDH1-mutant glioma. Neuro Oncol. (2025) 27:2023–34. doi: 10.1093/neuonc/noaf086, PMID: 40128633 PMC12448894

[59] Fan Q Kuang L Wang B Yin Y Dong Z Tian N . Multiple synergistic effects of the microglia membrane-bionic nanoplatform on mediate tumor microenvironment remodeling to amplify glioblastoma immunotherapy. ACS Nano. (2024) 18:14469–86. doi: 10.1021/acsnano.4c01253, PMID: 38770948

[60] Heiland DH Haaker G Delev D Mercas B Masalha W Heynckes S . Comprehensive analysis of PD-L1 expression in glioblastoma multiforme. Oncotarget. (2017) 8:42214–25. doi: 10.18632/oncotarget.15031, PMID: 28178682 PMC5522061

[61] Wang X Huang Z Wu Q Prager BC Mack SC Yang K . MYC-regulated mevalonate metabolism maintains brain tumor-initiating cells. Cancer Res. (2017) 77:4947–60. doi: 10.1158/0008-5472.Can-17-0114, PMID: 28729418 PMC5600855

[62] Bao S Wu Q McLendon RE Hao Y Shi Q Hjelmeland AB . Glioma stem cells promote radioresistance by preferential activation of the DNA damage response. Nature. (2006) 444:756–60. doi: 10.1038/nature05236, PMID: 17051156

[63] Calabrese C Poppleton H Kocak M Hogg TL Fuller C Hamner B . A perivascular niche for brain tumor stem cells. Cancer Cell. (2007) 11:69–82. doi: 10.1016/j.ccr.2006.11.020, PMID: 17222791

[64] Chen C Chen Z Luo R Tu W Long M Liang M . Endothelial USP11 drives VEGFR2 signaling and angiogenesis via PRDX2/c-MYC axis. Angiogenesis. (2025) 28:23. doi: 10.1007/s10456-025-09976-6, PMID: 40199774

[65] Bhat KPL Balasubramaniyan V Vaillant B Ezhilarasan R Hummelink K Hollingsworth F . Mesenchymal differentiation mediated by NF-κB promotes radiation resistance in glioblastoma. Cancer Cell. (2013) 24:331–46. doi: 10.1016/j.ccr.2013.08.001, PMID: 23993863 PMC3817560

[66] Hambardzumyan D Bergers G . Glioblastoma: defining tumor niches. Trends Cancer. (2015) 1:252–65. doi: 10.1016/j.trecan.2015.10.009, PMID: 27088132 PMC4831073

[67] Han S Liu Y Cai SJ Qian M Ding J Larion M . IDH mutation in glioma: molecular mechanisms and potential therapeutic targets. Br J Cancer. (2020) 122:1580–9. doi: 10.1038/s41416-020-0814-x, PMID: 32291392 PMC7250901

[68] Pirozzi CJ Yan H . The implications of IDH mutations for cancer development and therapy. Nat Rev Clin Oncol. (2021) 18:645–61. doi: 10.1038/s41571-021-00521-0, PMID: 34131315

[69] Sang Y Hou Y Cheng R Zheng L Alvarez AA Hu B . Targeting PDGFRα-activated glioblastoma through specific inhibition of SHP-2-mediated signaling. Neuro Oncol. (2019) 21:1423–35. doi: 10.1093/neuonc/noz107, PMID: 31232447 PMC6827835

[70] Mamer SB Chen S Weddell JC Palasz A Wittenkeller A Kumar M . Discovery of high-affinity PDGF-VEGFR interactions: redefining RTK dynamics. Sci Rep. (2017) 7:16439. doi: 10.1038/s41598-017-16610-z, PMID: 29180757 PMC5704011

[71] Klemm F Maas RR Bowman RL Kornete M Soukup K Nassiri S . Interrogation of the microenvironmental landscape in brain tumors reveals disease-specific alterations of immune cells. Cell. (2020) 181:1643–1660.e1617. doi: 10.1016/j.cell.2020.05.007, PMID: 32470396 PMC8558904

[72] Okada H Weller M Huang R Finocchiaro G Gilbert MR Wick W . Immunotherapy response assessment in neuro-oncology: a report of the RANO working group. Lancet Oncol. (2015) 16:e534–42. doi: 10.1016/s1470-2045(15)00088-1, PMID: 26545842 PMC4638131

[73] Woroniecka K Chongsathidkiet P Rhodin K Kemeny H Dechant C Farber SH . T-cell exhaustion signatures vary with tumor type and are severe in glioblastoma. Clin Cancer Res. (2018) 24:4175–86. doi: 10.1158/1078-0432.Ccr-17-1846, PMID: 29437767 PMC6081269

[74] Lim M Xia Y Bettegowda C Weller M . Current state of immunotherapy for glioblastoma. Nat Rev Clin Oncol. (2018) 15:422–42. doi: 10.1038/s41571-018-0003-5, PMID: 29643471

[75] Nikiforova MN Wald AI Melan MA Roy S Zhong S Hamilton RL . Targeted next-generation sequencing panel (GlioSeq) provides comprehensive genetic profiling of central nervous system tumors. Neuro Oncol. (2016) 18:379–87. doi: 10.1093/neuonc/nov289, PMID: 26681766 PMC4767245

[76] Kamran N Kadiyala P Saxena M Candolfi M Li Y Moreno-Ayala MA . Immunosuppressive myeloid cells’ Blockade in the glioma microenvironment enhances the efficacy of immune-stimulatory gene therapy. Mol Ther. (2017) 25:232–48. doi: 10.1016/j.ymthe.2016.10.003, PMID: 28129117 PMC5363306

[77] Zhang H Zhou Y Cui B Liu Z Shen H . Novel insights into astrocyte-mediated signaling of proliferation, invasion and tumor immune microenvironment in glioblastoma. BioMed Pharmacother. (2020) 126:110086. doi: 10.1016/j.biopha.2020.110086, PMID: 32172060

[78] Zhang X Rao A Sette P Deibert C Pomerantz A Kim WJ . IDH mutant gliomas escape natural killer cell immune surveillance by downregulation of NKG2D ligand expression. Neuro Oncol. (2016) 18:1402–12. doi: 10.1093/neuonc/now061, PMID: 27116977 PMC5035522

[79] Yeung JT Hamilton RL Ohnishi K Ikeura M Potter DM Nikiforova MN . LOH in the HLA class I region at 6p21 is associated with shorter survival in newly diagnosed adult glioblastoma. Clin Cancer Res. (2013) 19:1816–26. doi: 10.1158/1078-0432.CCR-12-2861, PMID: 23401227 PMC3618546

[80] Arvanitis CD Ferraro GB Jain RK . The blood-brain barrier and blood-tumour barrier in brain tumours and metastases. Nat Rev Cancer. (2020) 20:26–41. doi: 10.1038/s41568-019-0205-x, PMID: 31601988 PMC8246629

[81] Ellingson BM Bendszus M Sorensen AG Pope WB . Emerging techniques and technologies in brain tumor imaging. Neuro Oncol. (2014) 16 Suppl 7:vii12–23. doi: 10.1093/neuonc/nou221, PMID: 25313234 PMC4195527

[82] Wu D Chen Q Chen X Han F Chen Z Wang Y . The blood-brain barrier: structure, regulation, and drug delivery. Signal Transduct Target Ther. (2023) 8:217. doi: 10.1038/s41392-023-01481-w, PMID: 37231000 PMC10212980

[83] Wang Z Zhang H Xu S Liu Z Cheng Q . The adaptive transition of glioblastoma stem cells and its implications on treatments. Signal Transduct Target Ther. (2021) 6:124. doi: 10.1038/s41392-021-00491-w, PMID: 33753720 PMC7985200

[84] Tome-Garcia J Tejero R Nudelman G Yong RL Sebra R Wang H . Prospective isolation and comparison of human germinal matrix and glioblastoma EGFR(+) populations with stem cell properties. Stem Cell Rep. (2017) 8:1421–9. doi: 10.1016/j.stemcr.2017.03.019, PMID: 28434940 PMC5425658

[85] Hosseinalizadeh H Rahmati M Ebrahimi A O’Connor RS . Current status and challenges of vaccination therapy for glioblastoma. Mol Cancer Ther. (2023) 22:435–46. doi: 10.1158/1535-7163.Mct-22-0503, PMID: 36779991 PMC10155120

[86] Qin EY Cooper DD Abbott KL Lennon J Nagaraja S Mackay A . Neural precursor-derived pleiotrophin mediates subventricular zone invasion by glioma. Cell. (2017) 170:845–859.e819. doi: 10.1016/j.cell.2017.07.016, PMID: 28823557 PMC5587159

[87] Hollingsworth RE Jansen K . Turning the corner on therapeutic cancer vaccines. NPJ Vaccines. (2019) 4:7. doi: 10.1038/s41541-019-0103-y, PMID: 30774998 PMC6368616

[88] Haen SP Löffler MW Rammensee HG Brossart P . Towards new horizons: characterization, classification and implications of the tumour antigenic repertoire. Nat Rev Clin Oncol. (2020) 17:595–610. doi: 10.1038/s41571-020-0387-x, PMID: 32572208 PMC7306938

[89] Ilyas S Yang JC . Landscape of tumor antigens in T cell immunotherapy. J Immunol. (2015) 195:5117–22. doi: 10.4049/jimmunol.1501657, PMID: 26589749 PMC4656134

[90] Liu EK Sulman EP Wen PY Kurz SC . Novel therapies for glioblastoma. Curr Neurol Neurosci Rep. (2020) 20:19. doi: 10.1007/s11910-020-01042-6, PMID: 32445058

[91] Chen Z Wang X Yan Z Zhang M . Identification of tumor antigens and immune subtypes of glioma for mRNA vaccine development. Cancer Med. (2022) 11:2711–26. doi: 10.1002/cam4.4633, PMID: 35285582 PMC9249984

[92] Mendez-Gomez HR DeVries A Castillo P von Roemeling C Qdaisat S Stover BD . RNA aggregates harness the danger response for potent cancer immunotherapy. Cell. (2024) 187:2521–2535.e2521. doi: 10.1016/j.cell.2024.04.003, PMID: 38697107 PMC11767857

[93] Karimi-Sani I Molavi Z Naderi S Mirmajidi SH Zare I Naeimzadeh Y . Personalized mRNA vaccines in glioblastoma therapy: from rational design to clinical trials. J Nanobiotechnol. (2024) 22:601. doi: 10.1186/s12951-024-02882-x, PMID: 39367418 PMC11453023

[94] Bao R Spranger S Hernandez K Zha Y Pytel P Luke JJ . Immunogenomic determinants of tumor microenvironment correlate with superior survival in high-risk neuroblastoma. J Immunother Cancer. (2021) 9:e002417. doi: 10.1136/jitc-2021-002417, PMID: 34272305 PMC8287618

[95] De Groot AS Moise L Terry F Gutierrez AH Hindocha P Richard G . Better epitope discovery, precision immune engineering, and accelerated vaccine design using immunoinformatics tools. Front Immunol. (2020) 11:442. doi: 10.3389/fimmu.2020.00442, PMID: 32318055 PMC7154102

[96] Diao L Liu M . Rethinking antigen source: cancer vaccines based on whole tumor cell/tissue lysate or whole tumor cell. Adv Sci (Weinh). (2023) 10:e2300121. doi: 10.1002/advs.202300121, PMID: 37254712 PMC10401146

[97] Gondhowiardjo SA Handoko Jayalie VF Apriantoni R Barata AR Senoaji F . Tackling resistance to cancer immunotherapy: what do we know? Molecules. (2020) 25:4096. doi: 10.3390/molecules25184096, PMID: 32911646 PMC7570938

[98] Conforti A Salvatori E Lione L Compagnone M Pinto E Shorrock C . Linear DNA amplicons as a novel cancer vaccine strategy. J Exp Clin Cancer Res. (2022) 41:195. doi: 10.1186/s13046-022-02402-5, PMID: 35668533 PMC9169303

[99] Gao L Zhang A Yang F Du W . Immunotherapeutic strategies for head and neck squamous cell carcinoma (HNSCC): current perspectives and future prospects. Vaccines (Basel). (2022) 10:1272. doi: 10.3390/vaccines10081272, PMID: 36016159 PMC9416402

[100] Pudjihartono M Perry JK Print C O’Sullivan JM Schierding W . Interpretation of the role of germline and somatic non-coding mutations in cancer: expression and chromatin conformation informed analysis. Clin Epigenet. (2022) 14:120. doi: 10.1186/s13148-022-01342-3, PMID: 36171609 PMC9520844

[101] Capietto AH Jhunjhunwala S Pollock SB Lupardus P Wong J Hänsch L . Mutation position is an important determinant for predicting cancer neoantigens. J Exp Med. (2020) 217:e20190179. doi: 10.1084/jem.20190179, PMID: 31940002 PMC7144530

[102] Lione L Salvatori E Petrazzuolo A Massacci A Maggio R Confroti A . Antitumor efficacy of a neoantigen cancer vaccine delivered by electroporation is influenced by microbiota composition. Oncoimmunology. (2021) 10:1898832. doi: 10.1080/2162402x.2021.1898832, PMID: 33796408 PMC7993125

[103] Jiang T Zhu J Jiang S Chen Z Xu P Gong R . Targeting lncRNA DDIT4-AS1 Sensitizes Triple Negative Breast Cancer to Chemotherapy via Suppressing of Autophagy. Adv Sci (Weinh). (2023) 10:e2207257. doi: 10.1002/advs.202207257, PMID: 37096846 PMC10265098

[104] Rui Y Wilson DR Choi J Varanasi M Sanders K Karlsson J . Carboxylated branched poly(β-amino ester) nanoparticles enable robust cytosolic protein delivery and CRISPR-Cas9 gene editing. Sci Adv. (2019) 5:eaay3255. doi: 10.1126/sciadv.aay3255, PMID: 31840076 PMC6897553

[105] Zaki NM Tirelli N . Gateways for the intracellular access of nanocarriers: a review of receptor-mediated endocytosis mechanisms and of strategies in receptor targeting. Expert Opin Drug Deliv. (2010) 7:895–913. doi: 10.1517/17425247.2010.501792, PMID: 20629604

[106] Su T Liu X Lin S Cheng F Zhu G . Ionizable polymeric nanocarriers for the codelivery of bi-adjuvant and neoantigens in combination tumor immunotherapy. Bioact Mater. (2023) 26:169–80. doi: 10.1016/j.bioactmat.2023.02.016, PMID: 36883121 PMC9982230

[107] Awate S Babiuk LA Mutwiri G . Mechanisms of action of adjuvants. Front Immunol. (2013) 4:114. doi: 10.3389/fimmu.2013.00114, PMID: 23720661 PMC3655441

[108] Pulendran B A PS O’Hagan DT . Emerging concepts in the science of vaccine adjuvants. Nat Rev Drug Discov. (2021) 20:454–75. doi: 10.1038/s41573-021-00163-y, PMID: 33824489 PMC8023785

[109] Pashine A Valiante NM Ulmer JB . Targeting the innate immune response with improved vaccine adjuvants. Nat Med. (2005) 11:S63–68. doi: 10.1038/nm1210, PMID: 15812492

[110] Hailemichael Y Dai Z Jaffarzad N Ye Y Medina MA Huang XF . Persistent antigen at vaccination sites induces tumor-specific CD8^+^ T cell sequestration, dysfunction and deletion. Nat Med. (2013) 19:465–72. doi: 10.1038/nm.3105, PMID: 23455713 PMC3618499

[111] Chiang CL Kandalaft LE Coukos G . Adjuvants for enhancing the immunogenicity of whole tumor cell vaccines. Int Rev Immunol. (2011) 30:150–82. doi: 10.3109/08830185.2011.572210, PMID: 21557641

[112] Didierlaurent AM Morel S Lockman L Giannini SL Bisteau M Carlsen H . AS04, an aluminum salt- and TLR4 agonist-based adjuvant system, induces a transient localized innate immune response leading to enhanced adaptive immunity. J Immunol. (2009) 183:6186–97. doi: 10.4049/jimmunol.0901474, PMID: 19864596

[113] Everson RG Hugo W Sun L Antonios J Lee A Ding L . TLR agonists polarize interferon responses in conjunction with dendritic cell vaccination in Malignant glioma: a randomized phase II Trial. Nat Commun. (2024) 15:3882. doi: 10.1038/s41467-024-48073-y, PMID: 38719809 PMC11078958

[114] Alentorn A Marie Y Carpentier C Boisselier B Giry M Labussière M . Prevalence, clinico-pathological value, and co-occurrence of PDGFRA abnormalities in diffuse gliomas. Neuro Oncol. (2012) 14:1393–403. doi: 10.1093/neuonc/nos217, PMID: 23074200 PMC3480267

[115] Assanah MC Bruce JN Suzuki SO Chen A Goldman JE Canoll P . PDGF stimulates the massive expansion of glial progenitors in the neonatal forebrain. Glia. (2009) 57:1835–47. doi: 10.1002/glia.20895, PMID: 19533602

[116] Chen D Zuo D Luan C Liu M Na M Ran L . Glioma cell proliferation controlled by ERK activity-dependent surface expression of PDGFRA. PloS One. (2014) 9:e87281. doi: 10.1371/journal.pone.0087281, PMID: 24489888 PMC3906156

[117] Zou H Feng R Huang Y Tripodi J Najfeld V Tsankova NM . Double minute amplification of mutant PDGF receptor α in a mouse glioma model. Sci Rep. (2015) 5:8468. doi: 10.1038/srep08468, PMID: 25683249 PMC4329559

[118] Wang H Diaz AK Shaw TI Li Y Niu M Cho JH . Deep multiomics profiling of brain tumors identifies signaling networks downstream of cancer driver genes. Nat Commun. (2019) 10:3718. doi: 10.1038/s41467-019-11661-4, PMID: 31420543 PMC6697699

[119] Hermanson M Funa K Hartman M Claesson-Welsh L Heldin CH Westermark B . Platelet-derived growth factor and its receptors in human glioma tissue: expression of messenger RNA and protein suggests the presence of autocrine and paracrine loops. Cancer Res. (1992) 52:3213–9. 1317261

[120] Snuderl M Fazlollahi L Le LP Nitta M Zhelyazkova BH Davidson CJ . Mosaic amplification of multiple receptor tyrosine kinase genes in glioblastoma. Cancer Cell. (2011) 20:810–7. doi: 10.1016/j.ccr.2011.11.005, PMID: 22137795

[121] Szerlip NJ Pedraza A Chakravarty D Azim M McGuire J Fang Y . Intratumoral heterogeneity of receptor tyrosine kinases EGFR and PDGFRA amplification in glioblastoma defines subpopulations with distinct growth factor response. Proc Natl Acad Sci U S A. (2012) 109:3041–6. doi: 10.1073/pnas.1114033109, PMID: 22323597 PMC3286976

[122] Ahluwalia MS Reardon DA Abad AP Curry WT Wong ET Figel SA . Phase IIa study of surVaxM plus adjuvant temozolomide for newly diagnosed glioblastoma. J Clin Oncol. (2023) 41:1453–65. doi: 10.1200/jco.22.00996, PMID: 36521103 PMC9995096

[123] Platten M Bunse L Wick A Bunse T Le Cornet L Harting I . A vaccine targeting mutant IDH1 in newly diagnosed glioma. Nature. (2021) 592:463–8. doi: 10.1038/s41586-021-03363-z, PMID: 33762734 PMC8046668

[124] Sampson JH Aldape KD Archer GE Coan A Desjardins A Friedman AH . Greater chemotherapy-induced lymphopenia enhances tumor-specific immune responses that eliminate EGFRvIII-expressing tumor cells in patients with glioblastoma. Neuro Oncol. (2011) 13:324–33. doi: 10.1093/neuonc/noq157, PMID: 21149254 PMC3064599

[125] Sampson JH Heimberger AB Archer GE Aldape KD Friedman AH Friedman HS . Immunologic escape after prolonged progression-free survival with epidermal growth factor receptor variant III peptide vaccination in patients with newly diagnosed glioblastoma. J Clin Oncol. (2010) 28:4722–9. doi: 10.1200/jco.2010.28.6963, PMID: 20921459 PMC3020702

[126] Schuster J Lai RK Recht LD Reardon DA Paleologos NA Groves MD . multicenter trial of rindopepimut (CDX-110) in newly diagnosed glioblastoma: the ACT III study. Neuro Oncol. (2015) 17:854–61. doi: 10.1093/neuonc/nou348, PMID: 25586468 PMC4483122

[127] Weller M Butowski N Tran DD Recht LD Lim M Hirte H . Rindopepimut with temozolomide for patients with newly diagnosed, EGFRvIII-expressing glioblastoma (ACT IV): a randomised, double-blind, international phase 3 trial. Lancet Oncol. (2017) 18:1373–85. doi: 10.1016/s1470-2045(17)30517-x, PMID: 28844499

[128] Bloch O Crane CA Fuks Y Kaur R Aghi MK Berger MS . Heat-shock protein peptide complex-96 vaccination for recurrent glioblastoma: a phase II, single-arm trial. Neuro Oncol. (2014) 16:274–9. doi: 10.1093/neuonc/not203, PMID: 24335700 PMC3895386

[129] Bloch O Lim M Sughrue ME Komotar RJ Abrahams JM O’Rourke DM . Autologous heat shock protein peptide vaccination for newly diagnosed glioblastoma: impact of peripheral PD-L1 expression on response to therapy. Clin Cancer Res. (2017) 23:3575–84. doi: 10.1158/1078-0432.Ccr-16-1369, PMID: 28193626 PMC5511566

[130] Liau LM Ashkan K Brem S Campian JL Trusheim JE Iwamoto FM . Association of autologous tumor lysate-loaded dendritic cell vaccination with extension of survival among patients with newly diagnosed and recurrent glioblastoma: A phase 3 prospective externally controlled cohort trial. JAMA Oncol. (2023) 9:112–21. doi: 10.1001/jamaoncol.2022.5370, PMID: 36394838 PMC9673026

[131] Phuphanich S Raizer J Chamberlain M Canelos P Narwal R Hong S . Phase II study of MEDI-575, an anti-platelet-derived growth factor-α antibody, in patients with recurrent glioblastoma. J Neurooncol. (2017) 131:185–91. doi: 10.1007/s11060-016-2287-6, PMID: 27844311

[132] Wagner AJ Kindler H Gelderblom H Schöffski P Bauer S Hohenberger P . A phase II study of a human anti-PDGFRα monoclonal antibody (olaratumab, IMC-3G3) in previously treated patients with metastatic gastrointestinal stromal tumors. Ann Oncol. (2017) 28:541–6. doi: 10.1093/annonc/mdw659, PMID: 28426120 PMC5391707

[133] Lassman AB Pugh SL Gilbert MR Aldape KD Geinoz S Beumer JH . Phase 2 trial of dasatinib in target-selected patients with recurrent glioblastoma (RTOG 0627). Neuro Oncol. (2015) 17:992–8. doi: 10.1093/neuonc/nov011, PMID: 25758746 PMC5762006

[134] Sautter L Hofheinz R Tuettenberg J Grimm M Vajkoczy P Groden C . Open-label phase II evaluation of imatinib in primary inoperable or incompletely resected and recurrent glioblastoma. Oncology. (2020) 98:16–22. doi: 10.1159/000502483, PMID: 31514200

[135] von Mehren M Heinrich MC Shi H Iannazzo S Mankoski R Dimitrijević S . Clinical efficacy comparison of avapritinib with other tyrosine kinase inhibitors in gastrointestinal stromal tumors with PDGFRA D842V mutation: a retrospective analysis of clinical trial and real-world data. BMC Cancer. (2021) 21:291. doi: 10.1186/s12885-021-08013-1, PMID: 33740926 PMC7976710

[136] Louveau A Harris TH Kipnis J . Revisiting the mechanisms of CNS immune privilege. Trends Immunol. (2015) 36:569–77. doi: 10.1016/j.it.2015.08.006, PMID: 26431936 PMC4593064

[137] Wu Q Berglund AE Etame AB . The impact of epigenetic modifications on adaptive resistance evolution in glioblastoma. Int J Mol Sci. (2021) 22:8324. doi: 10.3390/ijms22158324, PMID: 34361090 PMC8347012

[138] Li W Graeber MB . The molecular profile of microglia under the influence of glioma. Neuro Oncol. (2012) 14:958–78. doi: 10.1093/neuonc/nos116, PMID: 22573310 PMC3408253

[139] Ross JL Velazquez Vega J Plant A MacDonald TJ Becher OJ Hambardzumyan D . Tumour immune landscape of paediatric high-grade gliomas. Brain. (2021) 144:2594–609. doi: 10.1093/brain/awab155, PMID: 33856022 PMC8536940

[140] Singh K Hotchkiss KM Mohan AA Reedy JL Sampson JH Khasraw M . For whom the T cells troll? Bispecific T-cell engagers in glioblastoma. J Immunother Cancer. (2021) 9:e003679. doi: 10.1136/jitc-2021-003679, PMID: 34795007 PMC8603282

[141] Li F Chen S Yu J Gao Z Sun Z Yi Y . Interplay of m(6) A and histone modifications contributes to temozolomide resistance in glioblastoma. Clin Transl Med. (2021) 11:e553. doi: 10.1002/ctm2.553, PMID: 34586728 PMC8441140

[142] Chen Q Jin J Li P Wang X Wang Q . Navigating glioma complexity: the role of abnormal signaling pathways in shaping future therapies. Biomedicines. (2025) 13:759. doi: 10.3390/biomedicines13030759, PMID: 40149733 PMC11940491

[143] Georgescu MM Islam MZ Li Y Circu ML Traylor J Notarianni CM . Global activation of oncogenic pathways underlies therapy resistance in diffuse midline glioma. Acta Neuropathol Commun. (2020) 8:111. doi: 10.1186/s40478-020-00992-9, PMID: 32680567 PMC7367358

[144] Kristoffersen K Villingshøj M Poulsen HS Stockhausen MT . Level of Notch activation determines the effect on growth and stem cell-like features in glioblastoma multiforme neurosphere cultures. Cancer Biol Ther. (2013) 14:625–37. doi: 10.4161/cbt.24595, PMID: 23792644 PMC3742492

[145] Xu A Wang X Luo J Zhou M Yi R Huang T . Overexpressed P75CUX1 promotes EMT in glioma infiltration by activating β-catenin. Cell Death Dis. (2021) 12:157. doi: 10.1038/s41419-021-03424-1, PMID: 33542188 PMC7862635

[146] Guo T Wu C Zhang J Yu J Li G Jiang H . Dual blockade of EGFR and PI3K signaling pathways offers a therapeutic strategy for glioblastoma. Cell Commun Signal. (2023) 21:363. doi: 10.1186/s12964-023-01400-0, PMID: 38115126 PMC10729576

[147] Ishikawa E Yamamoto T Matsumura A . Prospect of immunotherapy for glioblastoma: tumor vaccine, immune checkpoint inhibitors and combination therapy. Neurol Med Chir (Tokyo). (2017) 57:321–30. doi: 10.2176/nmc.nmc.ra.2016-0334, PMID: 28539528 PMC5566705

[148] Zahm CD Moseman JE Delmastro LE M DG . PD-1 and LAG-3 blockade improve anti-tumor vaccine efficacy. Oncoimmunology. (2021) 10:1912892. doi: 10.1080/2162402x.2021.1912892, PMID: 33996265 PMC8078506

[149] Antonios JP Soto H Everson RG Orpilla J Moughon D Shin N . PD-1 blockade enhances the vaccination-induced immune response in glioma. JCI Insight. (2016) 1:e87059. doi: 10.1172/jci.insight.87059, PMID: 27453950 PMC4951098

[150] Liu CJ Schaettler M Blaha DT Bowman-Kirigin JA Kobayashi DK Livingstone AJ . Treatment of an aggressive orthotopic murine glioblastoma model with combination checkpoint blockade and a multivalent neoantigen vaccine. Neuro Oncol. (2020) 22:1276–88. doi: 10.1093/neuonc/noaa050, PMID: 32133512 PMC7523441

[151] Awad MM Govindan R Balogh KN Spigel DR Garon EB Bushway ME . Personalized neoantigen vaccine NEO-PV-01 with chemotherapy and anti-PD-1 as first-line treatment for non-squamous non-small cell lung cancer. Cancer Cell. (2022) 40:1010–1026.e1011. doi: 10.1016/j.ccell.2022.08.003, PMID: 36027916

[152] Ott PA Hu-Lieskovan S Chmielowski B Govindan R Naing A Bhardwaj N . A phase ib trial of personalized neoantigen therapy plus anti-PD-1 in patients with advanced melanoma, non-small cell lung cancer, or bladder cancer. Cell. (2020) 183:347–362.e324. doi: 10.1016/j.cell.2020.08.053, PMID: 33064988

[153] Yarchoan M Gane EJ Marron TU Perales-Linares R Yan J Cooch N . Personalized neoantigen vaccine and pembrolizumab in advanced hepatocellular carcinoma: a phase 1/2 trial. Nat Med. (2024) 30:1044–53. doi: 10.1038/s41591-024-02894-y, PMID: 38584166 PMC11031401

[154] Blass E Ott PA . Advances in the development of personalized neoantigen-based therapeutic cancer vaccines. Nat Rev Clin Oncol. (2021) 18:215–29. doi: 10.1038/s41571-020-00460-2, PMID: 33473220 PMC7816749

[155] Keskin DB Anandappa AJ Sun J Tirosh I Mathewson ND Li S . Neoantigen vaccine generates intratumoral T cell responses in phase Ib glioblastoma trial. Nature. (2019) 565:234–9. doi: 10.1038/s41586-018-0792-9, PMID: 30568305 PMC6546179

[156] Ott PA Hu Z Keskin DB Shukla SA Sun J Bozym DJ . An immunogenic personal neoantigen vaccine for patients with melanoma. Nature. (2017) 547:217–21. doi: 10.1038/nature22991, PMID: 28678778 PMC5577644

[157] Sahin U Derhovanessian E Miller M Kloke BP Simon P Löwer M . Personalized RNA mutanome vaccines mobilize poly-specific therapeutic immunity against cancer. Nature. (2017) 547:222–6. doi: 10.1038/nature23003, PMID: 28678784

[158] Li J Guo Q Xing R . Construction and validation of an immune infiltration-related risk model for predicting prognosis and immunotherapy response in low grade glioma. BMC Cancer. (2023) 23:727. doi: 10.1186/s12885-023-11222-5, PMID: 37543576 PMC10403952

[159] Desjardins A Gromeier M Herndon JE 2nd Beaubier N Bolognesi DP Friedman AH . Recurrent glioblastoma treated with recombinant poliovirus. N Engl J Med. (2018) 379:150–61. doi: 10.1056/NEJMoa1716435, PMID: 29943666 PMC6065102

[160] Haddad AF Young JS Amara D Berger MS Raleigh DR Aghi MK . Mouse models of glioblastoma for the evaluation of novel therapeutic strategies. Neurooncol Adv. (2021) 3:vdab100. doi: 10.1093/noajnl/vdab100, PMID: 34466804 PMC8403483

[161] Morse MA Crosby EJ Force J Osada T Hobeika AC Hartman ZC . Clinical trials of self-replicating RNA-based cancer vaccines. Cancer Gene Ther. (2023) 30:803–11. doi: 10.1038/s41417-023-00587-1, PMID: 36765179 PMC9911953

[162] Rojas LA Sethna Z Soares KC Olcese C Pang N Patterson E . Personalized RNA neoantigen vaccines stimulate T cells in pancreatic cancer. Nature. (2023) 618:144–50. doi: 10.1038/s41586-023-06063-y, PMID: 37165196 PMC10171177

[163] Latzer P Zelba H Battke F Reinhardt A Shao B Bartsch O . A real-world observation of patients with glioblastoma treated with a personalized peptide vaccine. Nat Commun. (2024) 15:6870. doi: 10.1038/s41467-024-51315-8, PMID: 39127809 PMC11316744

[164] Sotoudeh H Shafaat O Bernstock JD Brooks MD Elsayed GA Chen JA . Artificial intelligence in the management of glioma: era of personalized medicine. Front Oncol. (2019) 9:768. doi: 10.3389/fonc.2019.00768, PMID: 31475111 PMC6702305

[165] Ren Y Yue Y Li X Weng S Xu H Liu L . Proteogenomics offers a novel avenue in neoantigen identification for cancer immunotherapy. Int Immunopharmacol. (2024) 142:113147. doi: 10.1016/j.intimp.2024.113147, PMID: 39270345

[166] Dapash M Hou D Castro B Lee-Chang C Lesniak MS . The interplay between glioblastoma and its microenvironment. Cells. (2021) 10:2257. doi: 10.3390/cells10092257, PMID: 34571905 PMC8469987

[167] Lakshmanachetty S Cruz-Cruz J Hoffmeyer E Cole AP Mitra SS . New insights into the multifaceted role of myeloid-derived suppressor cells (MDSCs) in high-grade gliomas: from metabolic reprograming, immunosuppression, and therapeutic resistance to current strategies for targeting MDSCs. Cells. (2021) 10:893. doi: 10.3390/cells10040893, PMID: 33919732 PMC8070707

[168] Arlen PM Madan RA Hodge JW Schlom J Gulley JL . Combining vaccines with conventional therapies for cancer. Update Cancer Ther. (2007) 2:33–9. doi: 10.1016/j.uct.2007.04.004, PMID: 17948067 PMC2034272

[169] Gulley JL Madan RA Arlen PM . Enhancing efficacy of therapeutic vaccinations by combination with other modalities. Vaccine. (2007) 25 Suppl 2:B89–96. doi: 10.1016/j.vaccine.2007.04.091, PMID: 17573164 PMC2062504

[170] Wheeler CJ Das A Liu G Yu JS Black KL . Clinical responsiveness of glioblastoma multiforme to chemotherapy after vaccination. Clin Cancer Res. (2004) 10:5316–26. doi: 10.1158/1078-0432.Ccr-04-0497, PMID: 15328167

[171] Wolfson B Franks SE Hodge JW . Stay on target: reengaging cancer vaccines in combination immunotherapy. Vaccines (Basel). (2021) 9:509. doi: 10.3390/vaccines9050509, PMID: 34063388 PMC8156017

[172] Wang S Jiang S Li X Huang H Qiu X Yu M . FGL2(172-220) peptides improve the antitumor effect of HCMV-IE1mut vaccine against glioblastoma by modulating immunosuppressive cells in the tumor microenvironment. Oncoimmunology. (2024) 13:2423983. doi: 10.1080/2162402x.2024.2423983, PMID: 39508842 PMC11542393

[173] Yang X Jiang S Liu F Li Z Liu W Zhang X . HCMV IE1/IE1mut therapeutic vaccine induces tumor regression via intratumoral tertiary lymphoid structure formation and peripheral immunity activation in glioblastoma multiforme. Mol Neurobiol. (2024) 61:5935–49. doi: 10.1007/s12035-024-03937-8, PMID: 38261253 PMC11249408

[174] Pellegatta S Valletta L Corbetta C Patanè M Zucca I Riccardi Sirtori F . Effective immuno-targeting of the IDH1 mutation R132H in a murine model of intracranial glioma. Acta Neuropathol Commun. (2015) 3:4. doi: 10.1186/s40478-014-0180-0, PMID: 25849072 PMC4359524

[175] Ochs K Ott M Bunse T Sahm F Bunse L Deumelandt K . K27M-mutant histone-3 as a novel target for glioma immunotherapy. Oncoimmunology. (2017) 6:e1328340. doi: 10.1080/2162402x.2017.1328340, PMID: 28811969 PMC5543817

[176] Ciesielski MJ Ahluwalia MS Munich SA Orton M Barone T Chanan-Khan A . Antitumor cytotoxic T-cell response induced by a survivin peptide mimic. Cancer Immunol Immunother. (2010) 59:1211–21. doi: 10.1007/s00262-010-0845-x, PMID: 20422411 PMC4603658

[177] Trivedi V Yang C Klippel K Yegorov O von Roemeling C Hoang-Minh L . mRNA-based precision targeting of neoantigens and tumor-associated antigens in Malignant brain tumors. Genome Med. (2024) 16:17. doi: 10.1186/s13073-024-01281-z, PMID: 38268001 PMC10809449

[178] Do ASS Amano T Edwards LA Zhang L De Peralta-Venturina M Yu JS . CD133 mRNA-loaded dendritic cell vaccination abrogates glioma stem cell propagation in humanized glioblastoma mouse model. Mol Ther Oncolytics. (2020) 18:295–303. doi: 10.1016/j.omto.2020.06.019, PMID: 32728617 PMC7378271

[179] Zhu S Lv X Zhang X Li T Zang G Yang N . An effective dendritic cell-based vaccine containing glioma stem-like cell lysate and CpG adjuvant for an orthotopic mouse model of glioma. Int J Cancer. (2019) 144:2867–79. doi: 10.1002/ijc.32008, PMID: 30565657

[180] Saka M Amano T Kajiwara K Yoshikawa K Ideguchi M Nomura S . Vaccine therapy with dendritic cells transfected with Il13ra2 mRNA for glioma in mice. J Neurosurg. (2010) 113:270–9. doi: 10.3171/2009.9.Jns09708, PMID: 19895199

[181] Pollack IF Jakacki RI Butterfield LH Hamilton RL Panigrahy A Normolle DP . Immune responses and outcome after vaccination with glioma-associated antigen peptides and poly-ICLC in a pilot study for pediatric recurrent low-grade gliomas. Neuro Oncol. (2016) 18:1157–68. doi: 10.1093/neuonc/now026, PMID: 26984745 PMC4933485

[182] Rampling R Peoples S Mulholland PJ James A Al-Salihi O Twelves CJ . A cancer research UK first time in human phase I trial of IMA950 (Novel multipeptide therapeutic vaccine) in patients with newly diagnosed glioblastoma. Clin Cancer Res. (2016) 22:4776–85. doi: 10.1158/1078-0432.Ccr-16-0506, PMID: 27225692 PMC5026298

[183] Fenstermaker RA Ciesielski MJ Qiu J Yang N Frank CL Lee KP . Clinical study of a survivin long peptide vaccine (SurVaxM) in patients with recurrent Malignant glioma. Cancer Immunol Immunother. (2016) 65:1339–52. doi: 10.1007/s00262-016-1890-x, PMID: 27576783 PMC5069322

[184] Wang QT Nie Y Sun SN Lin T Han RJ Jiang J . Tumor-associated antigen-based personalized dendritic cell vaccine in solid tumor patients. Cancer Immunol Immunother. (2020) 69:1375–87. doi: 10.1007/s00262-020-02496-w, PMID: 32078016 PMC11027674

[185] Hilf N Kuttruff-Coqui S Frenzel K Bukur V Stevanović S Gouttefangeas C . Actively personalized vaccination trial for newly diagnosed glioblastoma. Nature. (2019) 565:240–5. doi: 10.1038/s41586-018-0810-y, PMID: 30568303

[186] Berneman ZN De Laere M Germonpré P Huizing MT Willemen Y Lion E . WT1-mRNA dendritic cell vaccination of patients with glioblastoma multiforme, Malignant pleural mesothelioma, metastatic breast cancer, and other solid tumors: type 1 T-lymphocyte responses are associated with clinical outcome. J Hematol Oncol. (2025) 18:9. doi: 10.1186/s13045-025-01661-x, PMID: 39849594 PMC11755790

[187] Bota DA Taylor TH Piccioni DE Duma CM LaRocca RV Kesari S . Phase 2 study of AV-GBM-1 (a tumor-initiating cell targeted dendritic cell vaccine) in newly diagnosed Glioblastoma patients: safety and efficacy assessment. J Exp Clin Cancer Res. (2022) 41:344. doi: 10.1186/s13046-022-02552-6, PMID: 36517865 PMC9749349

